# Real-world fracture risk, osteoporosis treatment status, and mortality of Japanese non-dialysis patients with chronic kidney disease stages G3–5

**DOI:** 10.1007/s10157-024-02562-y

**Published:** 2024-10-14

**Authors:** Yasuo Imanishi, Satsuki Taniuchi, Sho Kodama, Hisako Yoshida, Tetsuo Ito, Ryota Kawai, Naoki Okubo, Ayumi Shintani

**Affiliations:** 1 Department of Metabolism, Endocrinology and Molecular Medicine, Osaka Metropolitan University Graduate School of Medicine, 1-4-3 Asahimachi, Abeno-Ku, Osaka, 545-8585 Japan; 2 Department of Medical Statistics, Osaka Metropolitan University Graduate School of Medicine, 1-4-3 Asahimachi, Abeno-Ku, Osaka, 545-8585 Japan; 3 ASCA Primary Product Department, ASCA Business Division, Daiichi Sankyo Co., Ltd., 3-5-1 Nihonbashi Honcho, Chuo-Ku, Tokyo, 103-8426 Japan; 4 Oncology Medical Science Department II, Medical Affairs Division, Daiichi Sankyo Co., Ltd., 3-5-1 Nihonbashi Honcho, Chuo-Ku, Tokyo, 103-8426 Japan; 5 Data Intelligence Department, Global DX, Daiichi Sankyo Co., Ltd., 1-2-58 Hiromachi, Shinagawa-Ku, Tokyo, 140-8710 Japan

**Keywords:** Bone mineral density, CKD, Fracture risk, GFR, Osteoporosis

## Abstract

**Background:**

Few studies have investigated fracture risk and mortality in a Japanese chronic kidney disease (CKD) stages G3–5 population using a large-scale clinical database.

**Methods:**

This retrospective cohort study extracted data from 1 April 2008 to 30 April 2023. A single age–sex-matched control without CKD was matched with each non-dialysis CKD (estimated glomerular filtration rate < 60 mL/min/1.73 m^2^) patient. The incidences of all and hip fractures and all-cause mortality after the index date were calculated.

**Results:**

Among 76,598 (38,299 per group) individuals matched, the incidence of all fractures did not differ between the CKD and control groups (5.7% vs 5.8%; hazard ratio [HR] 1.022 [95% confidence interval CI 0.952–1.098], *P* = 0.542). The CKD group had higher risk of hip fracture than the control group (incidence of hip fracture, 1.7% vs 1.3%; HR 1.415 [95% CI 1.234–1.622], *P* < 0.001). Multivariable regression analysis showed an increased risk for hip fracture in the CKD vs control groups, and a greater difference in this risk was observed with younger age. Osteoporosis treatment and bone mineral density (BMD) measurements were 10.0% and 5.3% in the CKD group and 4.4% and 4.4% in the control group, respectively. Mortality was also higher in the CKD group (HR 1.413 [95% CI 1.330–1.501], *P* < 0.001).

**Conclusions:**

Japanese patients with CKD had higher risk of hip fracture than those without. Treatment and BMD measurement for fracture are insufficient in Japanese patients with CKD, and more adequate management of fracture risk is needed.

**Supplementary Information:**

The online version contains supplementary material available at 10.1007/s10157-024-02562-y.

## Introduction

Globally, over 800 million people are affected by chronic kidney disease (CKD), with end-stage kidney disease having increased mortality rates [1, 2]. In Japan, the prevalence of CKD was estimated at about 13% of the adult population (13.3 million people) in 2005 [3], with a more recent study reporting that CKD prevalence was significantly higher in 2017 [4].

CKD is a lifestyle-related disease [5] and is considered a risk factor for stroke, cardiovascular disease, and hospitalization [2, 6, 7]. CKD also increases risk of fracture by four to six times that of age- and sex-matched controls without CKD [8–10]. Furthermore, patients with previous fractures have double the incidence of subsequent fractures, hampering the quality of life of patients and increasing mortality [11].

The effects of some pharmacotherapies on fractures in Japanese patients with CKD have been reported [12, 13], and several cross-sectional studies have reported a 1.3–7.1-fold increased risk of femoral and vertebral fractures in non-Japanese patients with renal impairment with an estimated glomerular filtration rate (eGFR) < 60 mL/min/1.73 m^2^ [14–18]. However, only one Japanese study has investigated the risk of new fracture onset in patients with CKD stages G3–5 (eGFR < 60 mL/min/1.73 m^2^), and this only included patients undergoing hemodialysis [12]. This has resulted in an evidence gap for Japanese patients with broader ranges of renal function (i.e., CKD stages G3–5). Therefore, it is important to assess epidemiological information on fracture risk and to detail the evidence of fractures occurring in CKD to understand the current status of medical care in Japanese patients with CKD.

The present study aimed to evaluate the risk of fracture and mortality, and the frequencies of osteoporosis treatments, in Japanese patients with CKD stages G3–5 in a real-world setting using a large-scale clinical database.

## Materials and methods

### Study design

This retrospective cohort study used data from anonymized electronic health insurance claims and diagnostic procedure combinations, linking routinely collected information for each patient, covering age, sex, height, weight, diagnosis based on International Classification of Diseases 10th Revision (ICD-10 codes), medical procedures, prescription medications, and survival status, spanning both outpatient and inpatient medical care held by Medical Data Vision (MDV; Tokyo, Japan) from 1 April 2008 to 30 April 2023. The MDV database comprises data from over 400 acute care hospitals, registering information for approximately 46 million patients, representing approximately 23% of all insurance claims in Japan [19]. So far, the MDV database has been used for several studies of CKD [20–22].

This study was conducted per the principles of the Declaration of Helsinki (as revised in 2013). The study was approved by Medical Corporation TOUKEIKAI Kitamachi Clinic Ethical Review Board (24 May 2023) and was registered (15 July 2023) in the University Hospital Medical Information Network (UMIN) Clinical Trials Registry under the identifier UMIN000051620. Informed consent was not obtained because the patient data are anonymized.

### Definition of CKD

CKD was defined according to the following: the patient (1) had at least two eGFR assessments < 60 mL/min/1.73 m^2^ at least 3 months apart; (2) had a diagnosis of CKD-related medical conditions (ICD-10 codes: N01–06, 11, 18, 26–28, Q60, 61) before the index date; and (3) aged ≥ 18 years at the time of the first eGFR measurement where the eGFR was < 60 mL/min/1.73 m^2^. The index date was defined as the time of the second eGFR measurement.

Patients with CKD who met any of the following criteria before the index date were excluded from this study: (1) received a definite diagnosis of cancer; (2) received a kidney transplant or renal dialysis; (3) had a diseases related to decreased bone density or was prescribed osteoporosis medication; (4) had a definite diagnosis of a fracture; and (5) had blood drawn in a specific department (such as an Emergency Room or Intensive Care Unit) on the index date.

### Selection of matched controls

For each patient with CKD, one control patient without CKD was selected when he/she met the following criteria as assessed at the index date of the intended CKD patient to be matched: (1) had at least one eGFR measurement ≥ 60 mL/min/1.73 m^2^ (the first measurement); (2) had at least one eGFR measurement ≥ 60 mL/min/1.73 m^2^ during a period from 3 months to 1 year after the first measurement of eGFR [23]; (3) was not identified as having CKD related diseases before the date of first measurement; (4) did not meet the above mentioned set of exclusion criteria as the CKD group; and (5) had the same age and sex as the CKD patient to be matched.

If the candidate control met the exclusion criteria, then matching was ceased, and a search was performed for a new candidate control for the CKD patient.

### Follow-up

Patients were censored at the earliest time when any of the following events whichever came first after the index date: (1) reached at the last observed day before no visit for > 3 years, or (2) reached at the final month of data provision to the MDV.

### Study outcomes

The primary endpoint was the incidence of fractures after the index date. The ICD-10 codes used as diagnosis of fractures are provided in Supplementary Table 1. The secondary endpoints included bone mineral density (BMD) measurements after the index date; osteoporosis and bone metabolism-related drug prescriptions after the index date; the incidence of all-cause mortality after the index date.

**Table 1:** Patient characteristics

	CKD (*n* = 38,299)	Control (*n* = 38,299)	SMD	Missing *n* (%)
Sex				
Male	25,671 (67.0)	25,671 (67.0)	< 0.001	0 (0.0)
Age (years)				
Mean ± SD	72.3 ± 12.7	72.3 ± 12.7	< 0.001	0 (0.0)
Median (Q1–Q3)	74 (65–82)	74 (65–82)		
< 65	8,998 (23.5)	8,998 (23.5)	< 0.001	
≥ 65	29,301 (76.5)	29,301 (76.5)		
Age group (years)				
10 s	12 (0.0)	12 (0.0)	< 0.001	0 (0.0)
20 s	104 (0.3)	104 (0.3)		
30 s	517 (1.3)	517 (1.3)		
40 s	1,777 (4.6)	1,777 (4.6)		
50 s	3,557 (9.3)	3,557 (9.3)		
60 s	7,464 (19.5)	7,464 (19.5)		
70 s	12,435 (32.5)	12,435 (32.5)		
80 s	10,651 (27.8)	10,651 (27.8)		
90 s	1,757 (4.6)	1,757 (4.6)		
≥ 100	25 (0.1)	25 (0.1)		
eGFR(mL/min/1.73 m^2^)				
Mean ± SD	33.8 ± 14.4	80.1 ± 19.4	2.708	0 (0.0)
Median (Q1–Q3)	34.4 (22.4–45.7)	75.4 (68.0–86.1)		
HbA1c (%)				
Mean ± SD	6.3 ± 1.0	6.4 ± 1.1	0.164	22,258 (29.1)
Median (Q1–Q3)	6.0 (5.6–6.6)	6.2 (5.7–6.9)		
Alkaline phosphatase (U/L)				
Mean ± SD	89.3 ± 41.8	88.8 ± 45.0	0.012	12,596 (16.4)
Median (Q1–Q3)	82 (66–102)	81 (66–100)		
Hospital scale (number of beds)				
≤ 199	3,695 (9.6)	4,693 (12.3)	0.140	0 (0.0)
200 to < 499	21,583 (56.4)	22,871 (59.7)		
≥ 500	13,021 (34.0)	10,735 (28.0)		
Complications				
Hypertension	26,973 (70.4)	19,061 (49.8)	0.432	0 (0.0)
Dyslipidemia	16,919 (44.2)	14,705 (38.4)	0.118	0 (0.0)
Hyperuricemia	13,356 (34.9)	2,886 (7.5)	0.710	0 (0.0)
Diabetes	16,739 (43.7)	15,975 (41.7)	0.040	0 (0.0)
Alcoholism	61 (0.2)	129 (0.3)	0.036	0 (0.0)
Rheumatoid arthritis	875 (2.3)	1,721 (4.5)	0.122	0 (0.0)
Dementia	1,565 (4.1)	2,525 (6.6)	0.112	0 (0.0)
Sleeping disorder	6,365 (16.6)	6,418 (16.8)	0.004	0 (0.0)
COPD	1,236 (3.2)	1,710 (4.5)	0.064	0 (0.0)
Drugs				
Glucocorticoid	1,567 (4.1)	2,106 (5.5)	0.066	0 (0.0)
Proton pump inhibitor	12,815 (33.5)	12,361 (32.3)	0.025	0 (0.0)
Hormonal therapy	67 (0.2)	65 (0.2)	0.001	0 (0.0)
Thiazoline	611 (1.6)	1,054 (2.8)	0.079	0 (0.0)
Beta-blocker	9,408 (24.6)	5,429 (14.2)	0.265	0 (0.0)
Loop diuretics	11,207 (29.3)	3,555 (9.3)	0.524	0 (0.0)
Heparin	5,634 (14.7)	4,273 (11.2)	0.106	0 (0.0)
Warfarin	2,884 (7.5)	1,605 (4.2)	0.143	0 (0.0)
Anti-anxiety drugs	7,683 (20.1)	7,780 (20.3)	0.006	0 (0.0)
Anticonvulsants	881 (2.3)	1,587 (4.1)	0.105	0 (0.0)
SSRI	257 (0.7)	317 (0.8)	0.018	0 (0.0)
Methotrexate	100 (0.3)	876 (2.3)	0.181	0 (0.0)
Calcimimetics	13 (0.0)	0 (0.0)	0.026	0 (0.0)
Phosphate binder	294 (0.8)	4 (0.0)	0.122	0 (0.0)

Data are *n* (%), mean ± SD or median (Q1–Q3)

*CKD* Chronic kidney disease, *COPD* Chronic obstructive pulmonary disease, *eGFR* estimated glomerular filtration rate, *HbA1c* glycosylated Hemoglobin, *Q* Quartile, *SD* Standard deviation, *SMD* Standardized mean difference, *SSRI* Selective serotonin reuptake inhibitor

### Statistical analysis

Because the study used an existing database, the sample size was not pre-specified. To describe patients’ characteristics at index date, frequencies and proportions were used for categorical variables; and mean and standard deviation (SD) and median and interquartile range were used for continuous variables. Standardized mean differences were used to assess differences between the CKD and the control groups.

The incidences of a fracture in the CKD and control groups were compared using Fine–Gray method where death was handled as a competing risk [24]. The covariates were added to each multivariable regression model to control for potential confounders. The covariates include age at the index date, sex, number of creatinine measurements in the year before the index date, comorbidities and prescribed medications. The list of comorbidities is provided in the Supplementary Text.

For assessing the effect of CKD over a wide age range, we test the hypothesis that the effect of CKD was null at any given level of age, the multivariable regression models included a cross-product term between CKD and age variables. Wald tests were conducted assessing difference between the full model including the cross-product terms and all covariates and the model excluding CKD main effect and the cross-product terms from the full model. In the multivariable analyses, non-linear effect of covariates of continuous variables were assessed using the cubic-spline method.

For the outcome of death, the Cox proportional hazards model adjusting for the above-described covariates was used to calculate the hazard ratio (HR) for death in the CKD group vs the control group. Effect modification (i.e., whether the effect of CKD on the risk of hip fracture was modified by levels of a covariate) was assessed using an interaction analysis where a cross-product term between the CKD group and a binary variable indicating a sub-group of patients was added to the above multivariable regression analyses. Forest plots were used to graphically depict the effect modifications.

The two-sided 5% significance level was used for all statistical inferences. No adjustment was made for multiple comparisons of *P*-values. All statistical analyses were conducted using R version 4.3.1 (The R Foundation for Statistical Computing, Vienna, Austria).

## Results

### Study population

For each of the CKD and control groups, 38,299 individuals were identified and used in the analysis.

Patient characteristics at the index date are shown in Table 1. In common with both groups, a mean ± SD age was 72.3 ± 12.7 years, 76.5% of patients were aged ≥ 65 years, and 67.0% of patients were male. The most common complications were hypertension, diabetes, and dyslipidemia, and the common concomitant medications were proton pump inhibitors, anti-anxiety drugs, beta-blockers, and loop diuretics.

The mean ± SD eGFR was 33.8 ± 14.4 mL/min/1.73 m^2^ in the CKD group and 80.1 ± 19.4 mL/min/1.73 m^2^ in the control group. The CKD group had a higher complication rate than the control group in hypertension (70.4% and 49.8%) and hyperuricemia (34.9% and 7.5%), and more received beta-blockers (24.6% and 14.2%) and loop diuretics (29.3% and 9.3%).

### Incidence of fractures

**Fig. 1:**
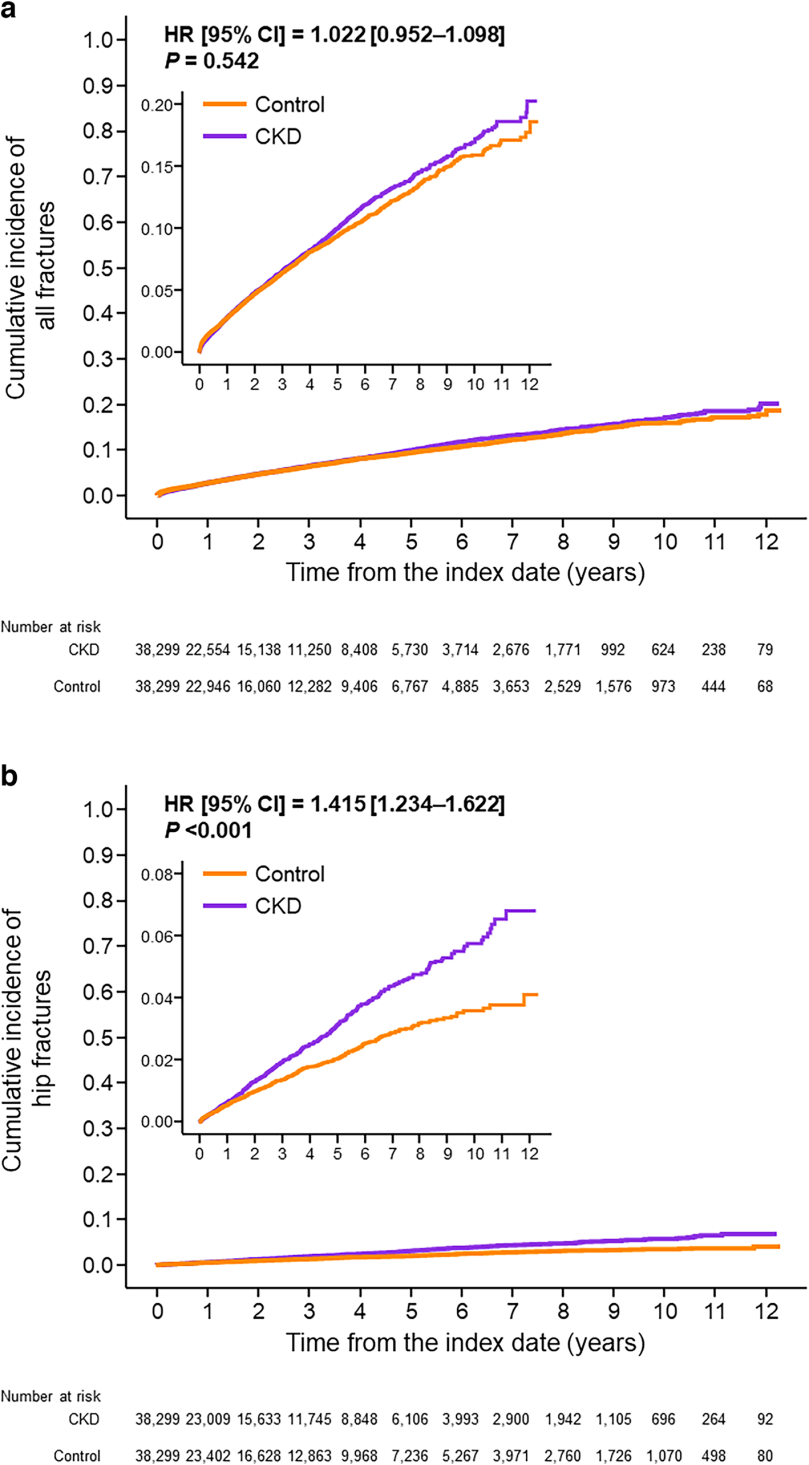
Incidences of **a** all fracture and **b** hip fracture after the index date. *CI* Confidence interval, *CKD* Chronic kidney disease, *HR* Hazard ratio calculated using the Fine–Gray model

The incidence of all fractures in the CKD and control groups was not significantly different (5.7% vs 5.8%; HR 1.022 [95% CI 0.952–1.098], *P* = 0.542; Table 2, Fig. 1a).

**Table 2:** Incidence of fracture

	*n*	Overall	Hip
Overall	76,598	4,402 (5.7)	1,132 (1.5)
Group			
Control	38,299	2,206 (5.8)	480 (1.3)
CKD	38,299	2,196 (5.7)	652 (1.7)
CKD stage			
G3a	10,111	386 (3.8)	77 (0.8)
G3b	12,763	688 (5.4)	183 (1.4)
G4–5	15,425	1,122 (7.3)	392 (2.5)
Age group (years)			
10 s	24	0 (0.0)	0 (0.0)
20 s	208	1 (0.5)	0 (0.0)
30 s	1,034	17 (1.6)	0 (0.0)
40 s	3,554	91 (2.6)	8 (0.2)
50 s	7,114	207 (2.9)	26 (0.4)
60 s	14,928	617 (4.1)	131 (0.9)
70 s	24,870	1,469 (5.9)	322 (1.3)
80 s	21,302	1,723 (8.1)	533 (2.5)
90 s	3,514	275 (7.8)	112 (3.2)
≥ 100	50	2 (4.0)	0 (0.0)
Sex			
Male	51,342	2,251 (4.4)	509 (1.0)
Female	25,256	2,251 (8.5)	623 (2.5)
Complications			
Hypertension	46,034	2,842 (6.2)	742 (1.6)
Dyslipidemia	31,624	1,788 (5.7)	425 (1.3)
Hyperuricemia	16,242	852 (5.2)	223 (1.4)
Diabetes	32,714	1,899 (5.8)	488 (1.5)
Alcoholism	190	10 (5.3)	1 (0.5)
Rheumatoid arthritis	2,596	190 (7.3)	41 (1.6)
Dementia	4,090	342 (8.4)	130 (3.2)
Sleeping disorder	12,783	1,006 (7.9)	255 (2.0)
COPD	2,946	194 (6.6)	41 (1.4)
Drugs			
Glucocorticoid	3,673	275 (7.5)	58 (1.6)
Proton pump inhibitor	25,176	1,633 (6.5)	437 (1.7)
Hormonal therapy	132	8 (6.1)	5 (3.8)
Thiazoline	1,665	138 (8.3)	26 (1.6)
Beta-blocker	14,837	785 (5.3)	227 (1.5)
Loop diuretics	14,762	1,149 (7.8)	361 (2.4)
Heparin	9,907	517 (5.2)	138 (1.4)
Warfarin	4,489	391 (8.7)	112 (2.5)
Anti-anxiety drugs	15,463	1,268 (8.2)	344 (2.2)
Anticonvulsants	2,468	189 (7.7)	63 (2.6)
SSRI	574	48 (8.4)	15 (2.6)
Methotrexate	976	60 (6.1)	9 (0.9)
Calcimimetics	13	0 (0.0)	0 (0.0)
Phosphate binder	298	18 (6.0)	2 (0.7)

Data are *n* (%)

*CKD* chronic kidney disease, *COPD* chronic obstructive pulmonary disease, *SSRI* selective serotonin reuptake inhibitor

Compared with the control group, the CKD group had a higher risk of hip fracture (incidence, 1.7% vs 1.3%; HR 1.415 [95% CI 1.234–1.622], *P* < 0.001; Table 2, Fig. 1b). However, the risk of vertebral fractures and non-vertebral fractures did not significantly differ between the groups (1.9% vs 2.1%; HR 0.962 [95% CI 0.854–1.083], *P* = 0.520; 4.0% vs 3.9%; HR 1.057 [95% CI 0.973–1.148], *P* = 0.188; Supplementary Table 2, Supplementary Figs. 1 and 2).

### Modification of effect of CKD vs control in the risk of hip fracture by age, sex, and eGFR

**Fig. 2:**
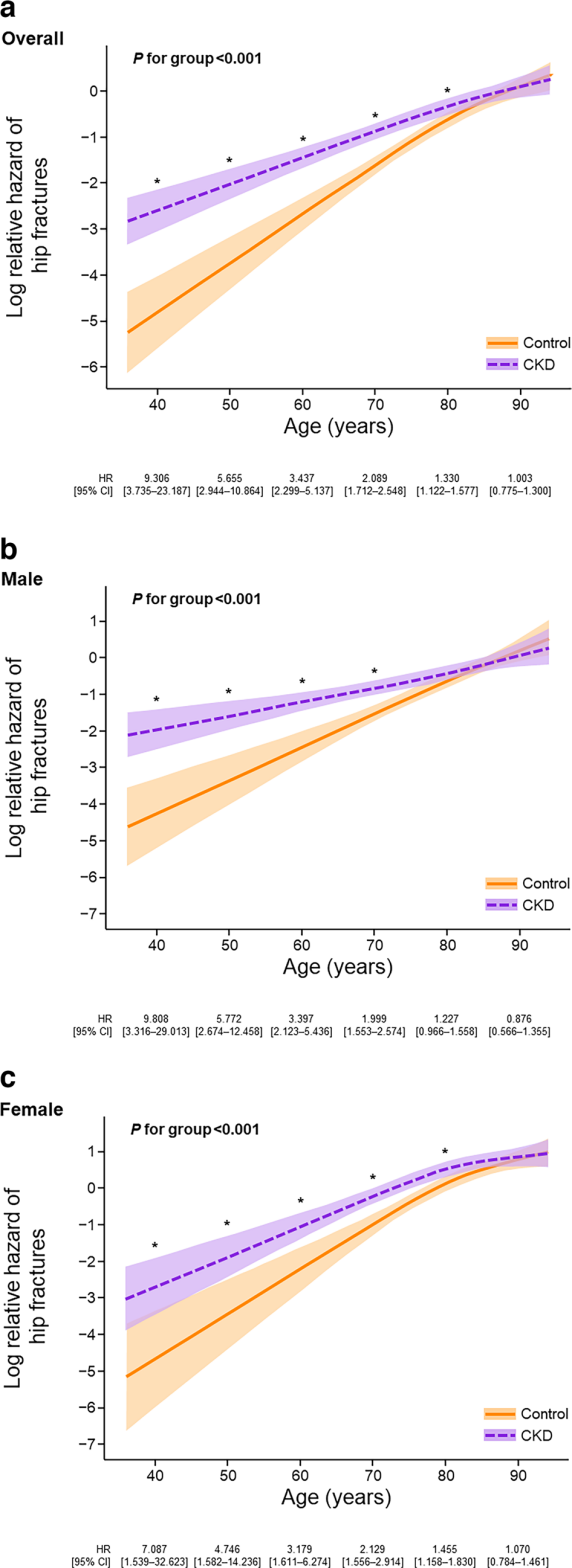
Multivariable regression analysis of hip fracture risk using age as covariate in **a** overall, **b** male, and **c** female populations. *P* for group < 0.05 rejects the null hypothesis indicating the HR of an event by CKD group = 1.0 for all age levels. * indicates a significant difference between the CKD and control groups. *CI* Confidence interval, *CKD* Chronic kidney disease, *HR* Hazard ratio calculated using the Fine–Gray model

Multivariable regression analysis for hip fracture risk showed that the CKD group had a higher risk of hip fracture at age levels ≤ 80 years (all *P* < 0.001); 40 years, HR 9.306 [95% CI 3.735–23.187]; 50 years, 5.655 [95% CI 2.944–10.864]; 60 years, 3.437 [95% CI 2.299–5.137]; 70 years, 2.089 [95% CI 1.712–2.548]; and 80 years, 1.330 [95% CI 1.122–1.577]. For patients aged 90 years, the effect was not statistically significant (HR 1.003 [95% CI 0.775–1.300]) (Fig. 2a). Similar tendencies, except for in males aged 80 years, were observed in both the male and female populations (Fig. 2b and c).

**Fig. 3:**
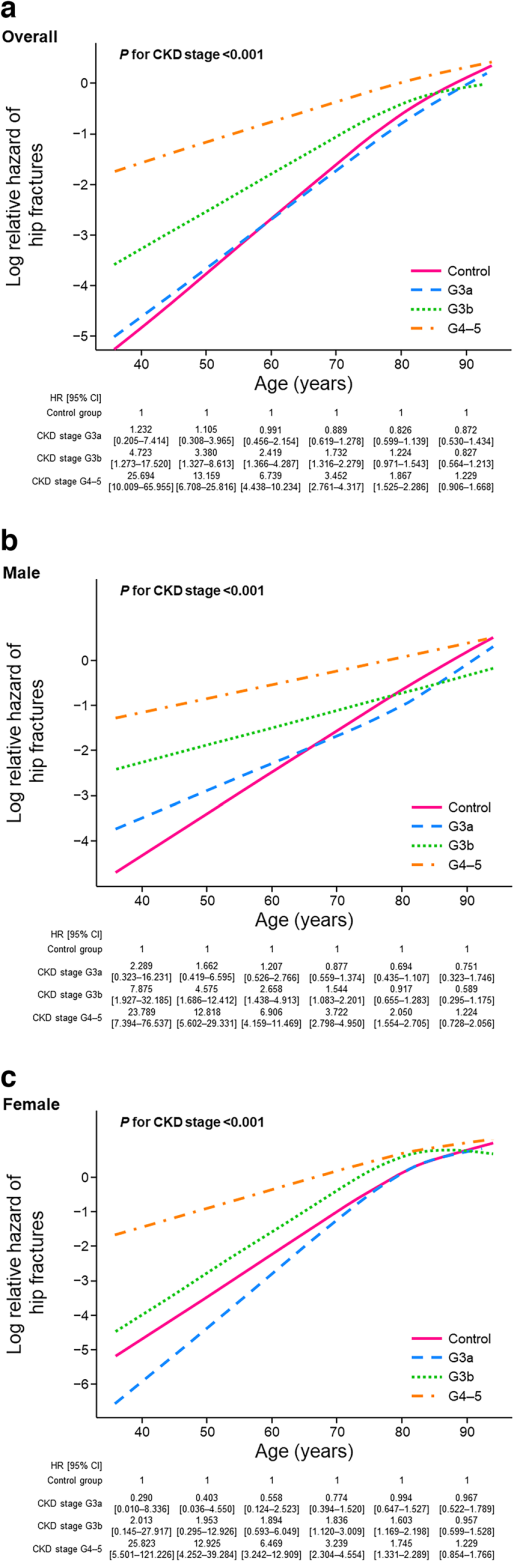
Multivariable regression analysis of hip fracture risk using age as covariate in **a** overall, **b** male, and **c** female populations in controls and patients with stage G3a, G3b, and G4–5 CKD. The HR was calculated for each age with controls as reference. *P* for CKD stage < 0.05 rejects the null hypothesis indicating the HR of an event by CKD stage = 1.0 for all age levels. *CI* Confidence interval, *CKD* Chronic kidney disease, *HR* Hazard ratio calculated using the Fine–Gray model

Figure 3 shows the risk of hip fracture by CKD stages at age levels in overall, male, and female populations. The difference in hip fracture risk between CKD stage G4–5 patients and controls was notable in younger patients, with CKD stages G4–5 patients in the 40, 50, 60, 70, and 80 year groups having significantly higher risks of hip fracture than controls (Fig. 3a). However, in the populations aged ≥ 90 years, the risk of hip fracture was similar between patients with CKD stages G4–5 and controls. Similar trends were observed irrespective of sex (Fig. 3b and c).

Incidences of hip fractures in the CKD vs control groups within each subgroup of patients are shown in Supplementary Fig. 3. Statistically significantly associated effect modifiers were older age (≥ 80 years vs < 80 years), dementia, and the use of anti-anxiety drugs or phosphate binders (*P* values for interaction < 0.05).

An increased risk of hip fracture with decreasing eGFR was also observed (*P* < 0.001) (Supplementary Fig. 4).

### Bone mineral density measurements, prescription osteoporosis medications and mortality

The BMD measurements rates after the index date were 5.3% and 4.4% in CKD and control groups, respectively (Table 3).

**Table 3:** Bone mineral density measurements and prescriptions for osteoporosis and bone metabolism-related drugs after the index date by CKD stage

	Control	CKD			
	(*n* = 38,299)	G3–5 (*n* = 38,299)	G3a (*n* = 10,111)	G3b (*n* = 12,763)	G4–5 (*n* = 15,425)
Bone mineral density measurements	1,699 (4.4)	2,020 (5.3)	431 (4.3)	542 (4.2)	1,047 (6.8)
Osteoporosis and bone metabolism-related drugs	1,697 (4.4)	3,821 (10.0)	477 (4.7)	833 (6.5)	2,511 (16.3)
SERMs	70 (0.2)	99 (0.3)	24 (0.2)	24 (0.2)	51 (0.3)
Bazedoxifene acetate	29 (0.1)	50 (0.1)	14 (0.1)	9 (0.1)	27 (0.2)
Raloxifene hydrochloride	44 (0.1)	60 (0.2)	11 (0.1)	15 (0.1)	34 (0.2)
Ipriflavone	1 (0.0)	2 (0.0)	0 (0.0)	1 (0.0)	1 (0.0)
Calcium drugs	75 (0.2)	89 (0.2)	13 (0.1)	28 (0.2)	48 (0.3)
Calcium hydrogen phosphate hydrate	1 (0.0)	0 (0.0)	0 (0.0)	0 (0.0)	0 (0.0)
Calcium L-aspartate hydrate	74 (0.2)	89 (0.2)	13 (0.1)	28 (0.2)	48 (0.3)
Calcitonin drugs	52 (0.1)	86 (0.2)	13 (0.1)	23 (0.2)	50 (0.3)
Elkatonin	52 (0.1)	86 (0.2)	13 (0.1)	23 (0.2)	50 (0.3)
Calcitonin (salmon)	0 (0.0)	0 (0.0)	0 (0.0)	0 (0.0)	0 (0.0)
Bisphosphonates	910 (2.4)	843 (2.2)	247 (2.4)	295 (2.3)	301 (2.0)
Alendronate (P.O.)	516 (1.3)	558 (1.5)	173 (1.7)	211 (1.7)	174 (1.1)
Ibandronate (P.O.)	33 (0.1)	27 (0.1)	8 (0.1)	13 (0.1)	6 (0.0)
Etidronate (P.O.)	0 (0.0)	0 (0.0)	0 (0.0)	0 (0.0)	0 (0.0)
Minodronic acid (P.O.)	182 (0.5)	151 (0.4)	45 (0.4)	52 (0.4)	54 (0.4)
Risedronate (P.O.)	209 (0.5)	111 (0.3)	37 (0.4)	39 (0.3)	35 (0.2)
Alendronate (I.V.)	11 (0.0)	17 (0.0)	5 (0.0)	4 (0.0)	8 (0.1)
Ibandronate (I.V.)	30 (0.1)	55 (0.1)	5 (0.0)	12 (0.1)	38 (0.2)
Zoledronate (I.V.)	17 (0.0)	5 (0.0)	4 (0.0)	1 (0.0)	0 (0.0)
Vitamin K_2_ drugs (menatetrenone)	34 (0.1)	65 (0.2)	11 (0.1)	16 (0.1)	38 (0.2)
Parathyroid hormone drugs	145 (0.4)	97 (0.3)	18 (0.2)	34 (0.3)	45 (0.3)
Abaloparatide acetate	0 (0.0)	0 (0.0)	0 (0.0)	0 (0.0)	0 (0.0)
Teriparatide acetate	74 (0.2)	49 (0.1)	7 (0.1)	16 (0.1)	26 (0.2)
Teriparatide (genetical recombination)	74 (0.2)	48 (0.1)	11 (0.1)	18 (0.1)	19 (0.1)
Female hormone drugs	62 (0.2)	38 (0.1)	13 (0.1)	15 (0.1)	10 (0.1)
Estradiol	2 (0.0)	0 (0.0)	0 (0.0)	0 (0.0)	0 (0.0)
Estriol	60 (0.2)	38 (0.1)	13 (0.1)	15 (0.1)	10 (0.1)
Anti-RANKL antibodies (denosumab)	154 (0.4)	123 (0.3)	26 (0.3)	52 (0.4)	45 (0.3)
Anti-sclerostin antibody drugs (romosozumab)	48 (0.1)	20 (0.1)	4 (0.0)	7 (0.1)	9 (0.1)
Active vitamin D_3_ drugs	886 (2.3)	3,001 (7.8)	225 (2.2)	556 (4.4)	2,220 (14.4)
Alfacalcidol	411 (1.1)	2,410 (6.3)	145 (1.4)	411 (3.2)	1,854 (12.0)
Eldecalcitol	536 (1.4)	402 (1.0)	91 (0.9)	143 (1.1)	168 (1.1)
Calcitriol	14 (0.0)	453 (1.2)	10 (0.1)	52 (0.4)	391 (2.5)

Data are *n* (%)

*CKD* chronic kidney disease, *I.V.* intravenous administration, *P.O.* oral administration, *RANKL* receptor activator of nuclear factor-kappa B ligand, *SERMs* selective estrogen receptor

Prescription osteoporosis medication status after the index date according to CKD stage and other characteristics are summarized in Table 3. Overall, prescription rates for osteoporosis medications were 10.0% and 4.4% in CKD and control groups, respectively. Regarding bisphosphonate, the use of oral alendronate decreased with increasing CKD stage. For active vitamin D_3_ preparations, the use of alfacalcidol and calcitriol increased with increasing CKD stage. For other medications, no differences by CKD stage existed.

**Fig. 4:**
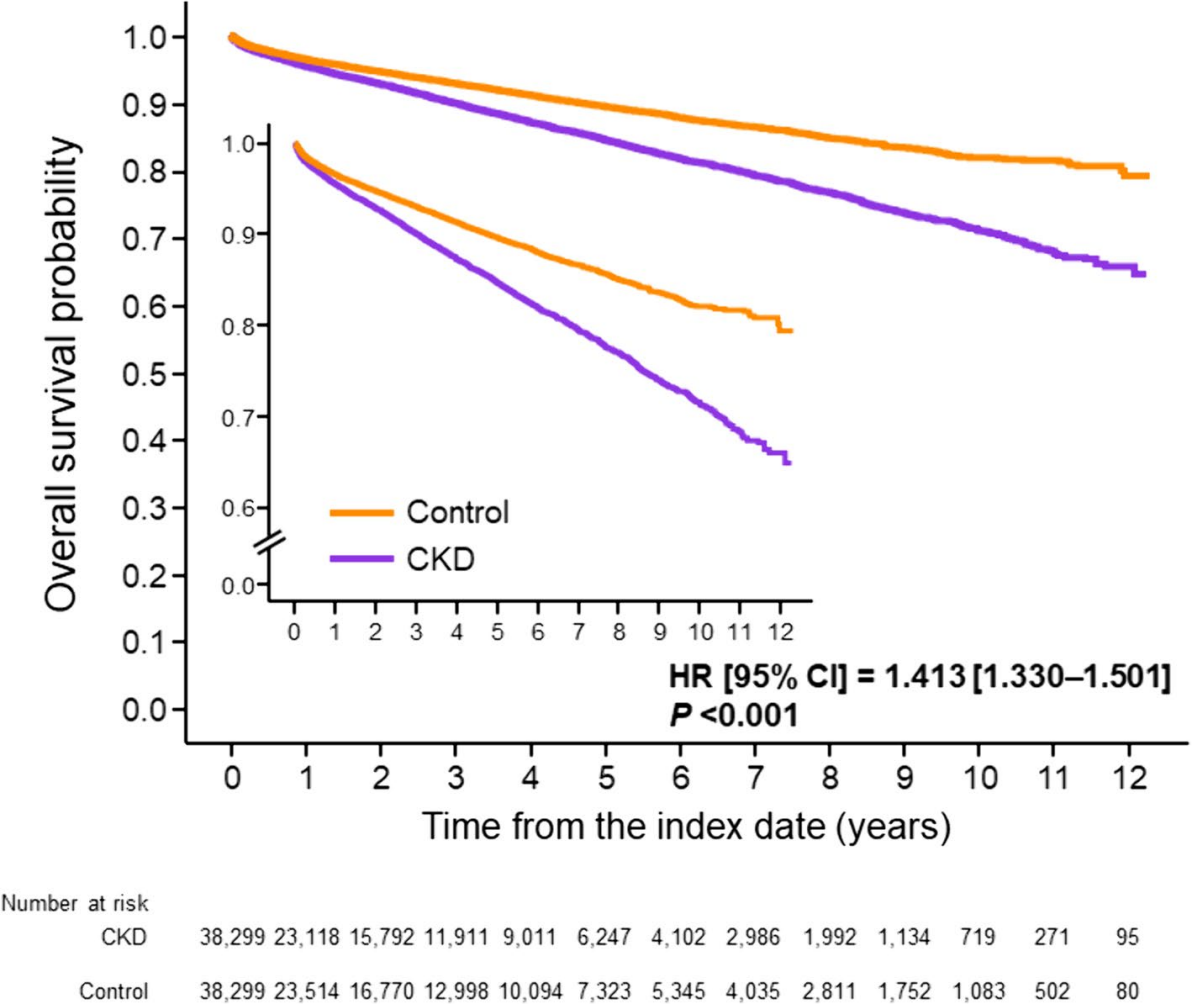
Incidence of all-cause mortality after the index date *CI* confidence interval, *CKD* chronic kidney disease, *HR* hazard ratio calculated using the Cox proportional hazards model

The incidence of all-cause mortality was higher in the CKD group vs the control group: HR 1.413 [95% CI 1.330–1.501, *P* < 0.001 (Fig. 4).

## Discussion

To the best of our knowledge, this retrospective database study is the first of its kind to investigate fracture risk, osteoporosis treatment status, and mortality in Japanese patients with CKD stages G3–5 (eGFR < 60 mL/min/1.73 m^2^) compared with control patients (eGFR ≥ 60 mL/min/1.73 m^2^). A previous Japanese study on fracture risk included only patients undergoing hemodialysis [12]. The present study addressed this evidence gap by identifying fracture risk for patients with a wide range of renal dysfunction (CKD stages G3–5).

This study found that the risk of all fractures was similar between the CKD and control groups, but hip fracture risk was significantly higher in the CKD group (1.415-fold increase). The increased hip fracture risk was more notable in patients ≤ 80 years old. Despite this increased hip fracture risk, most patients did not receive BMD measurements and osteoporosis medications. Additionally, the mortality risk was higher (1.413-fold increase) in the CKD group compared with the control group.

In the present study, the CKD group had a fracture risk of HR 1.022 (95% CI 0.952–1.098) for all fractures, HR 1.415 (95% CI 1.234–1.622) for hip fractures, HR 0.962 (95% CI 0.854–1.083) for vertebral fractures, and HR 1.057 (95% CI 0.973–1.148) for non-vertebral fractures. Hip fractures are expected to increase in CKD because the cortical bone deterioration increases due to cortical porosity partly caused by secondary hyperparathyroidism of uremia. [25]. Vertebral fractures are generally more common than other types of fractures, but were not significantly increased in the CKD group in this study. In addition, there were also no significant differences in the incidences of non-vertebral fractures, including hip fractures, between the groups in this study. Thus, it is likely that this was not a significant contributor to the total number of fractures. These results are similar to those of previous non-Japanese studies that found that hip fracture risk increased in patients with eGFR < 60 mL/min/1.73 m^2^ [15, 16, 18, 26, 27].

There are limited number of previous Japanese studies that can be compared with our study, however, our results were generally consistent with a study of patients with prior osteoporotic fracture, in which CKD grade 4/5 was associated with 1-year subsequent fracture risk (HR 1.30 [1.16–1.46]) [28]. Conversely, the fracture incidence observed in the present study was lower than in a previous Japanese study that included patients on hemodialysis (CKD stage G5D) [12]. In that study, the standardized incidence ratios of hip fractures were compared between hemodialysis patients vs the general population, with overall incidence and standardized incidence ratios of 7.57/1000 person-years and 6.2 [95% CI 5.7–6.8] in male patients and 17.43/1000 person-years and 4.9 [95% CI 4.6–5.3] in female patients, respectively [12]. Excluding patients with a history of kidney transplant, renal dialysis, decreased bone density, or osteoporosis medication prescriptions in our study may have resulted in a lower fracture incidence.

In this study, hip fracture risk increased with age regardless of CKD status and renal function. In addition, younger female patients had increased hip fracture risk in the CKD group vs the control group and this difference became notable with younger age, while the risk was similar between older (≥ 90 years) patients with and without CKD. This suggests that CKD may have a greater impact on hip fracture risk in younger patients, but in older populations the impact of factors other than CKD, such as patient age, may become more important. This finding is generally consistent with a previous study [16] that found that female patients with an eGFR ≤ 60 mL/min/1.73 m^2^, especially those with eGFR < 45 mL/min/1.73 m^2^ had higher risk of hip fracture (HR 2.32) than those with eGFR 45–59 mL/min/1.73 m^2^ (HR 1.57, *P* for trend = 0.02).

In this study, most patients did not undergo BMD measurements (CKD patients: 5.3%, controls: 4.4%) or receive osteoporosis medication (CKD patients: 10.0%, controls: 4.4%). According to the Japanese 2015 guidelines for prevention and treatment of osteoporosis [29], if an osteopenic patient with “no fragility fracture and BMD greater than 70% and less than 80% in young adult mean [YAM] value” has CKD (eGFR < 60 mL/min/1.73 m^2^), the initiation of pharmacological treatment for fracture risk should be considered. However, the timing of BMD measurement in patients with CKD is not mentioned in this guideline and is dependent on the judgment of the physician in clinical practice. Therefore, the results of this study may reflect the status of the guideline.

Although the populations were different, the BMD measurement rate in this study was similar to the osteoporosis screening (interview + bone mass measurement) rate of 5.3% reported in 2021 by the Japan Osteoporosis Foundation [30, 31]. The osteoporosis screening rate is calculated by dividing the number of persons aged 40, 45, 50, 55, 60, 65, and 70 years who underwent osteoporosis screening by the total Japanese female population in the same age categories. Conversely, the target osteoporosis screening rate set in 2032 in the Japanese Government’s Health Japan 21 guideline (the third stage, basic policy, and health promotion plan based on the Health Promotion Law of Japan) [32] is set at 15%, which is much higher than the BMD measurement rate observed in our study.

Japanese CKD practice guidelines [33] specify the use of osteoporosis medications for CKD patients already diagnosed with osteoporosis, but do not include a treatment policy for the prevention of osteoporosis onset associated with reduced renal function. Therefore, the rate of BMD measurements and osteoporosis treatment in CKD patients may be lower than is ideal. The Glucocorticoid-Induced Osteoporosis (GIOP) Practice Guidelines (2014) [34] recommend BMD measurements and the administration of osteoporosis medications for patients taking glucocorticoids, considering the risk of osteoporosis-related fractures. Before this 2014 guideline revision, the rate of bone densitometry in GIOP patients was 5.5% in 2011, slightly increasing to 6.6% in 2018, while the rate of osteoporosis medication prescriptions increased even more, from 40.0% to 51.8% at the same time points. These increases may be due to a change in the recommended requirement for starting GIOP treatment of bone density measurement in 2004 [35] to a scoring system in the 2014 GIOP guidelines. It is hoped that studies such as this one will increase awareness of the risks of fractures in CKD patients due to osteoporosis and that, in future, osteoporosis screening and treatment rates will increase in this patient population.

The risk of hip fracture in the CKD group was higher than in the control group in the present study, and if a hip fracture actually occurred, it would significantly reduce the quality of life of patients [11]. Therefore, patients with CKD stages G3–5 require more frequent or regular BMD measurements to start appropriate pharmacological treatment and prevent fractures. It should be noted that active vitamin D_3_ drugs, which had the highest prescription rate in our study, may have been used to prevent hypocalcemia and/or secondary hyperparathyroidism due to reduced renal function, rather than as a treatment for osteoporosis. In fact, the proportion of patients receiving such prescriptions increased in proportion to the progression of CKD stage. Therefore, the actual rate of prescription for the treatment of osteoporosis in this patient population may be even lower.

This study had some limitations and inherent risk of bias due to the data source and study design. The period for which pre-existing fractures were identifiable was limited to the period of data extraction. Fractures that occurred at other medical institutions were not able to be tracked. Thus, there is no information on treatment and prescription history prior to the visit, or visits to other hospitals, even during the follow-up period. The MDV database mainly comprises of data from relatively large acute care hospitals. The patients in this study were limited to those who attended acute care hospitals and had laboratory values recorded, which may have potential bias for patient selection, and the findings may not be generalizable because of the database composition. The control group was also enrolled from patients visiting the hospital, and thus was not representative of a healthy population. Another limitation of our study is that we did not assess parathyroid hormone values. The use of drugs such as teriparatide and abaloparatide may affect parathyroid hormone levels, persistently high levels of which promote bone resorption and affect fracture risk [36, 37]. Future analyses should include parathyroid hormone values to assess their influence on fracture events.

## Conclusions

This study fills an evidence gap by identifying fracture risk for Japanese patients with wide ranges of renal dysfunction by CKD stage. The incidence of all fractures was not higher in patients with CKD than those without, but the risk of hip fracture was higher in patients with CKD. In addition, most patients with CKD did not receive osteoporosis treatment or undergo BMD measurements. Hip fractures tend to confine patients to bed and may lead to disuse syndrome, which requires additional care. Therefore, more appropriate management of fracture risk in CKD patients is considered to be a future priority in CKD care.

## Supplementary Information

Below is the link to the electronic supplementary material.

Supplementary file1 (DOCX 365 KB)

Supplementary file2 (TIF 57 KB)

Supplementary file3 (TIF 61 KB)

Supplementary file4 (TIF 182 KB)

Supplementary file5 (TIF 42 KB)

## Data Availability

The deidentifed participant data and the study protocol will be shared on a request basis for up to 36 months after the publication of this article. Researchers who make the request should include a methodologically sound proposal on how the data will be used; the proposal may be reviewed by the responsible personnel at Daiichi Sankyo Co., Ltd., and the data requestors will need to sign a data access agreement. Please directly contact the corresponding author to request data sharing.

## References

[1] Kovesdy CP. Epidemiology of chronic kidney disease: an update 2022. Kidney Int Suppl. 2011;2022(12):7–11. 10.1016/j.kisu.2021.11.003 PMC9073222 35529086

[2] Go AS, Chertow GM, Fan D, et al. Chronic kidney disease and the risks of death, cardiovascular events, and hospitalization. N Engl J Med. 2004;351:1296–305. 15385656 10.1056/NEJMoa041031

[3] Imai E, Horio M, Watanabe T, et al. Prevalence of chronic kidney disease in the Japanese general population. Clin Exp Nephrol. 2009;13:621–30. 19513802 10.1007/s10157-009-0199-x

[4] Takeuchi M, Shinkawa K, Yanagita M, et al. Prevalence, recognition and management of chronic kidney disease in Japan: population-based estimate using a healthcare database with routine health checkup data. Clin Kidney J. 2021;14:2197–202. 34676073 10.1093/ckj/sfab016 PMC8528067

[5] Kanazawa I, Inaba M, Inoue D, et al. Executive summary of clinical practice guide on fracture risk in lifestyle diseases. J Bone Miner Metab. 2020;38:746–58. 32892240 10.1007/s00774-020-01149-3

[6] Masson P, Webster AC, Hong M, et al. Chronic kidney disease and the risk of stroke: a systematic review and meta-analysis. Nephrol Dial Transplant. 2015;30:1162–9. 25681099 10.1093/ndt/gfv009

[7] GBD Chronic Kidney Disease Collaboration. Global, regional, and national burden of chronic kidney disease, 1990–2017: a systematic analysis for the global burden of disease study 2017. Lancet. 2020;395:709–33. 32061315 10.1016/S0140-6736(20)30045-3 PMC7049905

[8] Naylor KL, McArthur E, Leslie WD, et al. The three-year incidence of fracture in chronic kidney disease. Kidney Int. 2014;86:810–8. 24429401 10.1038/ki.2013.547

[9] Evenepoel P, Cunningham J, Ferrari S, et al. European consensus statement on the diagnosis and management of osteoporosis in chronic kidney disease stages G4–G5D. Nephrol Dial Transplant. 2021;36:42–59. 33098421 10.1093/ndt/gfaa192

[10] Bellorin-Font E, Rojas E, Martin KJ. Bone disease in chronic kidney disease and kidney transplant. Nutrients. 2022;15:167. 36615824 10.3390/nu15010167 PMC9824497

[11] Bliuc D, Nguyen ND, Milch VE, et al. Mortality risk associated with low-trauma osteoporotic fracture and subsequent fracture in men and women. JAMA. 2009;301:513–21. 19190316 10.1001/jama.2009.50

[12] Wakasugi M, Kazama JJ, Taniguchi M, et al. Increased risk of hip fracture among Japanese hemodialysis patients. J Bone Miner Metab. 2013;31:315–21. 23292163 10.1007/s00774-012-0411-z

[13] Shukuri T, Nakai K, Tanaka S, et al. Angiotensin II receptor blockers and bone fracture in chronic kidney disease patients: the Fukuoka kidney disease Registry Study. Clin Exp Nephrol. 2023;27:919–27. 37498346 10.1007/s10157-023-02385-3

[14] Dukas L, Schacht E, Stähelin HB. In elderly men and women treated for osteoporosis a low creatinine clearance of <65 ml/min is a risk factor for falls and fractures. Osteoporos Int. 2005;16:1683–90. 15933802 10.1007/s00198-005-1903-7

[15] Nickolas TL, McMahon DJ, Shane E. Relationship between moderate to severe kidney disease and hip fracture in the United States. J Am Soc Nephrol. 2006;17:3223–32. 17005938 10.1681/ASN.2005111194

[16] Ensrud KE, Lui LY, Taylor BC, et al. Renal function and risk of hip and vertebral fractures in older women. Arch Intern Med. 2007;167:133–9. 17242313 10.1001/archinte.167.2.133

[17] Fried LF, Biggs ML, Shlipak MG, et al. Association of kidney function with incident hip fracture in older adults. J Am Soc Nephrol. 2007;18:282–6. 17167115 10.1681/ASN.2006050546

[18] LaCroix AZ, Lee JS, Wu L, et al. Cystatin-C, renal function, and incidence of hip fracture in postmenopausal women. J Am Geriatr Soc. 2008;56:1434–41. 18662213 10.1111/j.1532-5415.2008.01807.x PMC2891241

[19] Medical Data Vision. About MDV Database. Accessed 20 February 2024. http://www.mdv.co.jp/

[20] Matsuzaki T, Watanabe Y, Tanaka A, et al. Prognosis and incidence of infections in chronic kidney disease patients with membranous nephropathy enrolled in a large Japanese clinical claims database. BMC Nephrol. 2023;24:126. 37142947 10.1186/s12882-023-03190-6 PMC10161415

[21] Liyanage T, Toyama T, Hockham C, et al. Prevalence of chronic kidney disease in Asia: a systematic review and analysis. BMJ Glob Health. 2022;7:e007525. 35078812 10.1136/bmjgh-2021-007525 PMC8796212

[22] Kashihara N, Kohsaka S, Kanda E, et al. Hyperkalemia in real-world patients under continuous medical care in Japan. Kidney Int Rep. 2019;4:1248–60. 31517144 10.1016/j.ekir.2019.05.018 PMC6734103

[23] Matsuo S, Yasuda Y, Imai E, et al. Current status of estimated glomerular filtration rate (eGFR) equations for Asians and an approach to create a common eGFR equation. Nephrology (Carlton). 2010;15(Suppl 2):45–8. 20586948 10.1111/j.1440-1797.2010.01313.x

[24] Fine JP, Gray RJ. A proportional hazards model for the subdistribution of a competing risk. J Am Stat Assoc. 1999;94:496–509.

[25] Kidney Disease: Improving Global Outcomes (KDIGO) CKD-MBD Work Group. KDIGO. clinical practice guideline update for the diagnosis, evaluation, prevention, and treatment of chronic kidney disease-mineral and bone disorder (CKD-MBD). Kidney Int Suppl. 2017;2017(7):1–59. 10.1016/j.kisu.2017.04.001 PMC6340919 30675420

[26] Desbiens LC, Goupil R, Madore F, et al. Incidence of fractures in middle-aged individuals with early chronic kidney disease: A population-based analysis of CARTaGENE. Nephrol Dial Transplant. 2020;35:1712–21. 31951261 10.1093/ndt/gfz259

[27] Vilaca T, Salam S, Schini M, et al. Risks of hip and nonvertebral fractures in patients with CKD G3a–G5D: a systematic review and meta-analysis. Am J Kidney Dis. 2020;76:521–32. 32654892 10.1053/j.ajkd.2020.02.450

[28] Fujiwara S, Buchanan-Hughes A, Ng A, et al. Real-world evaluation of osteoporotic fractures using the Japan Medical Data Vision database. Osteoporos Int. 2022;33:2205–16. 35779100 10.1007/s00198-022-06472-1

[29] Japan Osteoporosis Society, The Japanese Society for Bone and Mineral Research, Japan Osteoporosis Foundation (eds). Japanese 2015 guidelines for prevention and treatment of osteoporosis. Tokyo: Life Science Publishing; 2015 (In Japanese).

[30] Japan Osteoporosis Foundation. Number and rate of osteoporosis screening. Accessed 10 June 2024. https://www.jpof.or.jp/Portals/0/pdf/screening_rate/screeningrate_2021.pdf (In Japanese).

[31] Japan Osteoporosis Foundation. Osteoporosis screening. Accessed 10 June 2024. https://www.jpof.or.jp/osteoporosis/inspection_treatment/tabid252.html (In Japanese).

[32] Ministry of Health, Labour and Welfare in Japan. Explanatory material for the promotion of Healthy Japan 21 (the third term) (part 2). Accessed 10 June 2024.https://www.mhlw.go.jp/content/001158871.pdf (In Japanese).

[33] Japanese Society of Nephrology. Essential points from evidence-based clinical practice guideline for chronic kidney disease 2023. Clin Exp Nephrol. 2024;8:473–95. 10.1007/s10157-024-02497-4 PMC11116248 38713253

[34] Suzuki Y, Nawata H, Soen S, et al. Guidelines on the management and treatment of glucocorticoid-induced osteoporosis of the Japanese Society for Bone and Mineral Research: 2014 update. J Bone Miner Metab. 2014;32:337–50. 24818875 10.1007/s00774-014-0586-6

[35] Nawata H, Soen S, Takayanagi R, et al. Subcommittee to study diagnostic criteria for glucocorticoid-induced osteoporosis. guidelines on the management and treatment of glucocorticoid-induced osteoporosis of the Japanese Society for Bone and Mineral Research (2004). J Bone Miner Metab. 2005;23:105–9. 15750687 10.1007/s00774-004-0596-x

[36] Komaba H, Imaizumi T, Hamano T, et al. Lower parathyroid hormone levels are associated with reduced fracture risk in Japanese hemodialysis patients. Kidney Int Rep. 2024. 10.1016/j.ekir.2024.07.008. 39430172 10.1016/j.ekir.2024.07.008 PMC11489479

[37] Yamamoto S, Jørgensen HS, Zhao J, et al. Alkaline phosphatase and parathyroid hormone levels: international variation and associations with clinical outcomes in the DOPPS. Kidney Int Rep. 2024;9:863–76. 38765600 10.1016/j.ekir.2024.01.002 PMC11101738

