# Supplementary material for: Real-world fracture risk, osteoporosis treatment status, and mortality of Japanese non-dialysis patients with chronic kidney disease stages G3–5
Source: Clin Exp Nephrol. 2024 Oct 14;29(2):236–47. doi: 10.1007/s10157-024-02562-y (PMC11828842; doi:10.1007/s10157-024-02562-y)
Supplement: Supplementary file 1 — Supplementary file1 (DOCX 365 KB) [file 10157_2024_2562_MOESM1_ESM.docx]

**Supplementary Materials**

**Supplementary Text**

The list of comorbidities includes a history of the following: hypertension, dyslipidemia, hyperuricemia, diabetes, alcohol dependence, rheumatoid arthritis, dementia, sleep disorders, and chronic obstructive pulmonary disease. The prescribed medications included as covariates were oral glucocorticoids, proton pump inhibitors, hormone replacement therapy drugs, thiazolidinedione antidiabetic drugs, oral beta-blockers, oral loop diuretics, heparin, warfarin, sleep aids, anxiolytics, antiepileptic drugs, selective serotonin reuptake inhibitors, methotrexate, calcium-sensing receptor agonists, and phosphate-binding agents.

**Supplementary Figures**

**Supplementary Fig. 1:** Incidence of vertebral fractures.


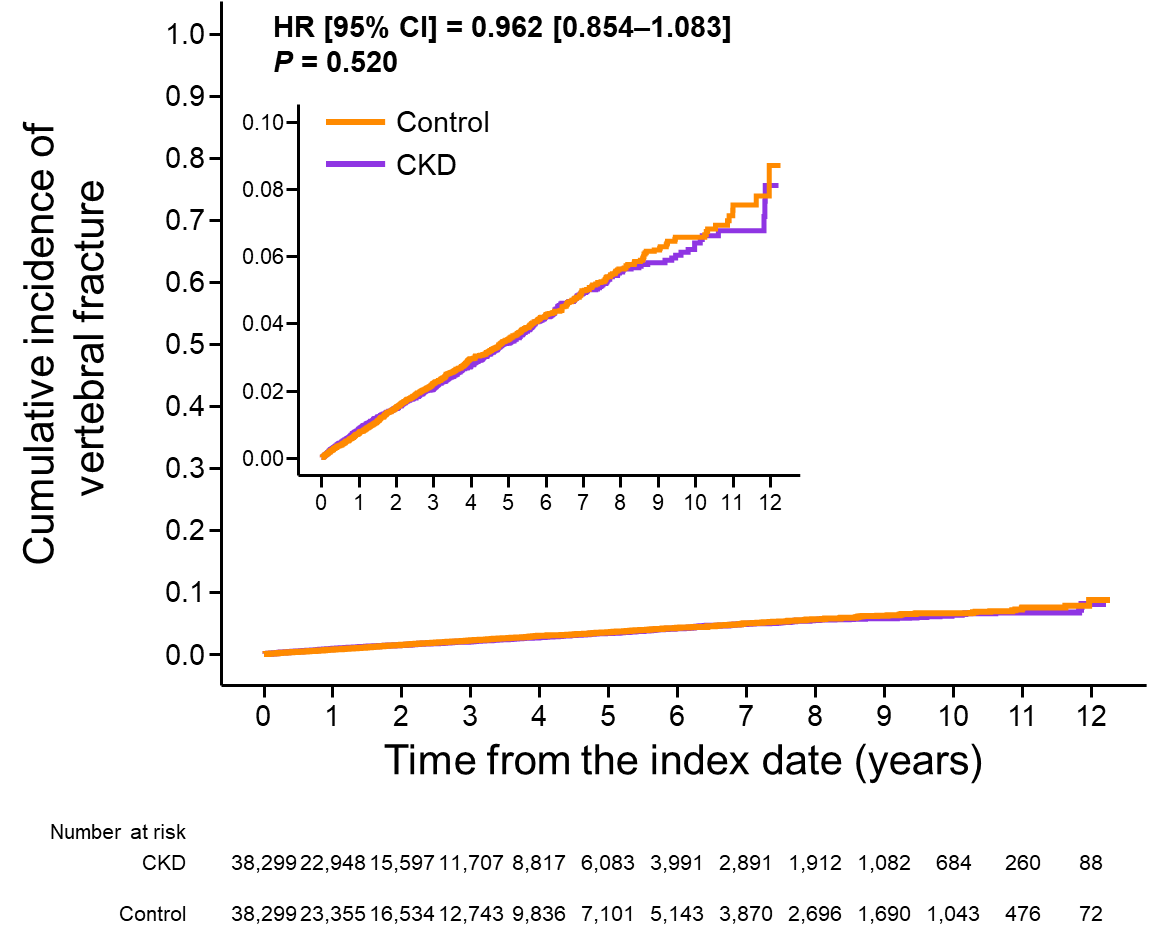


*CI* confidence interval, *CKD* chronic kidney disease, *HR* hazard ratio calculated using the Fine–Gray model

**Supplementary Fig. 2:** Incidence of non-vertebral fractures.


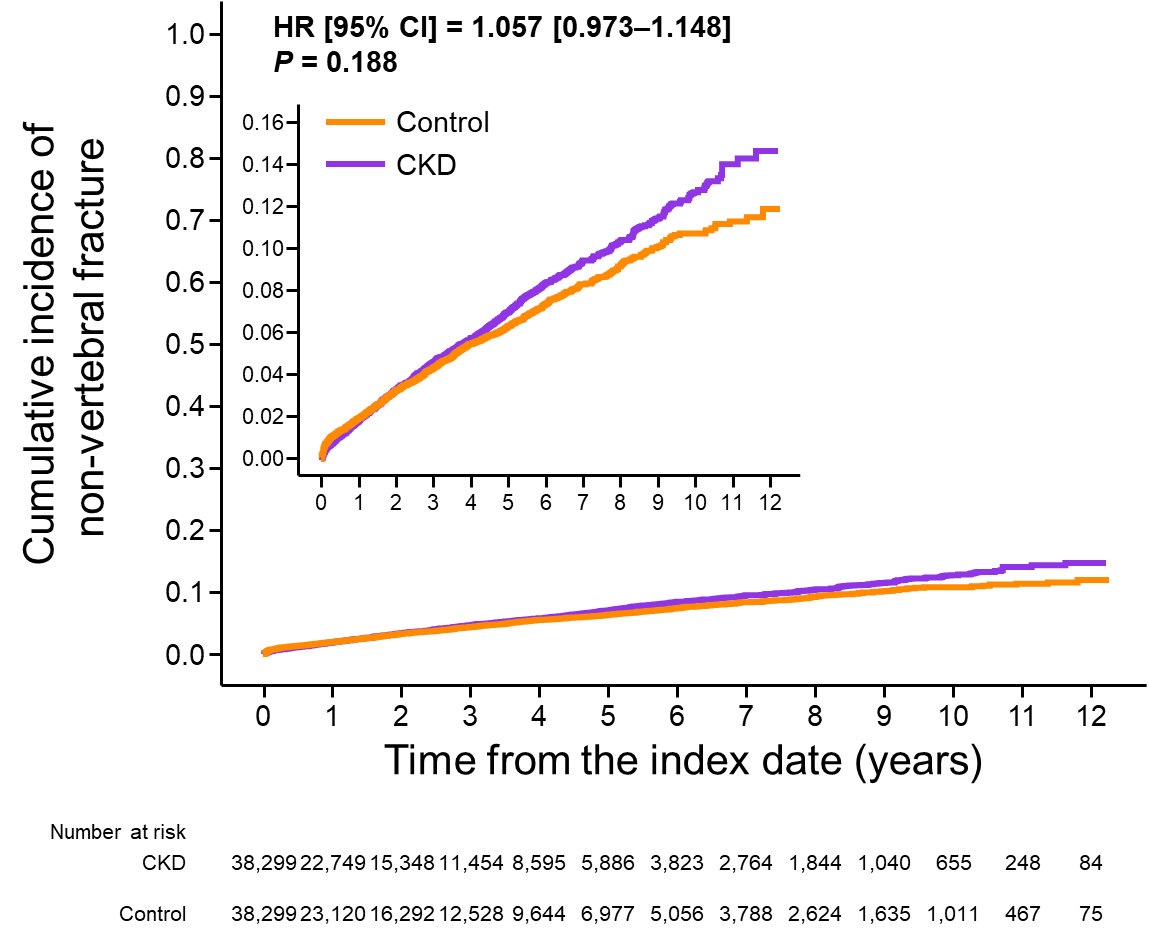


*CI* confidence interval, *CKD* chronic kidney disease, *HR* hazard ratio calculated using the Fine–Gray model

**Supplementary Fig. 3:** Multivariable analysis for hip fracture according to CKD and control group.


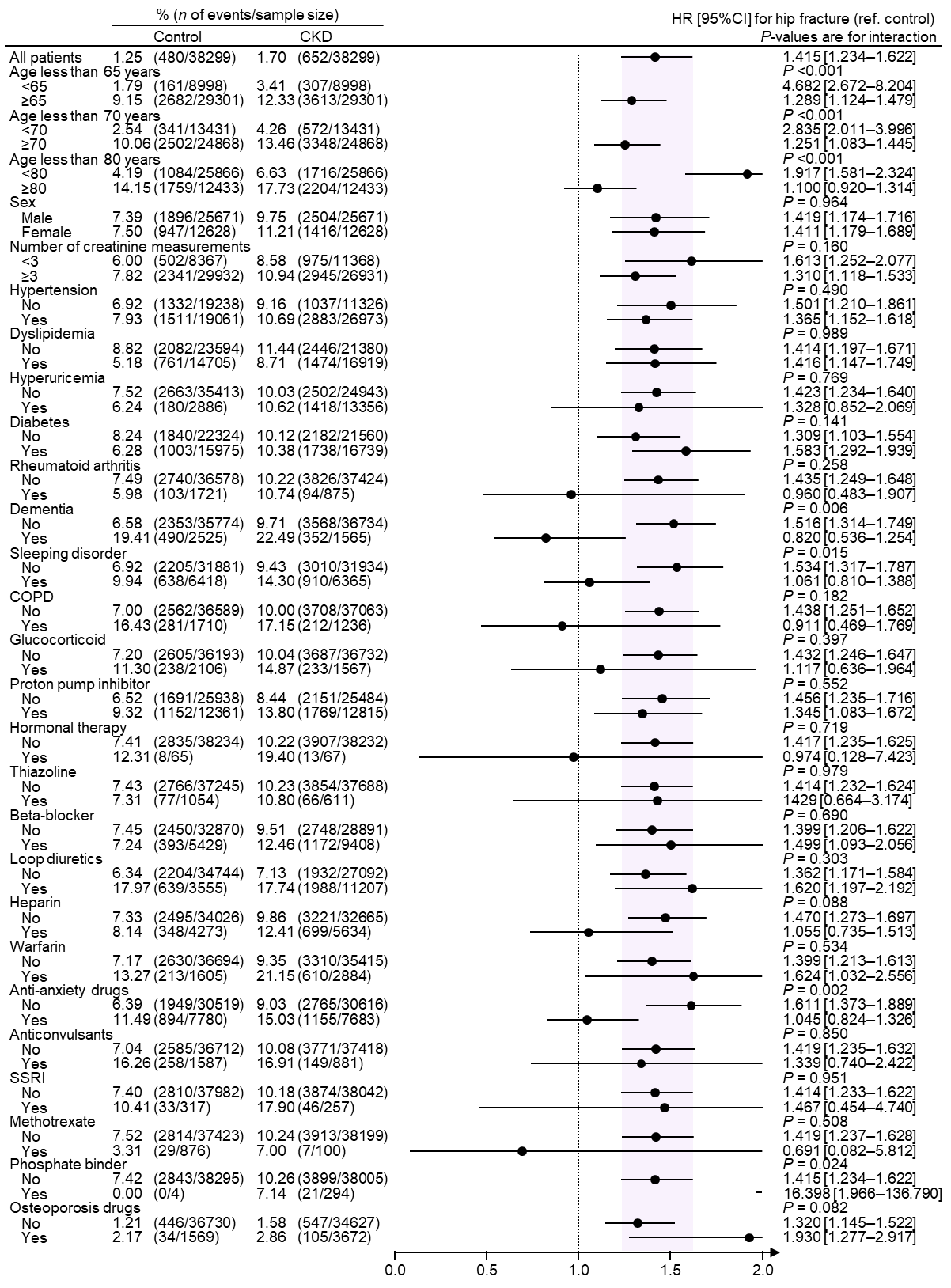


*CI* confidence interval, *CKD* chronic kidney disease, *COPD* chronic obstructive pulmonary disease, *HR* hazard ratio, *SSRI* selective serotonin reuptake inhibitor

**Supplementary Fig. 4:** Multivariable regression analysis for hip fracture risk using eGFR value as covariate.

*
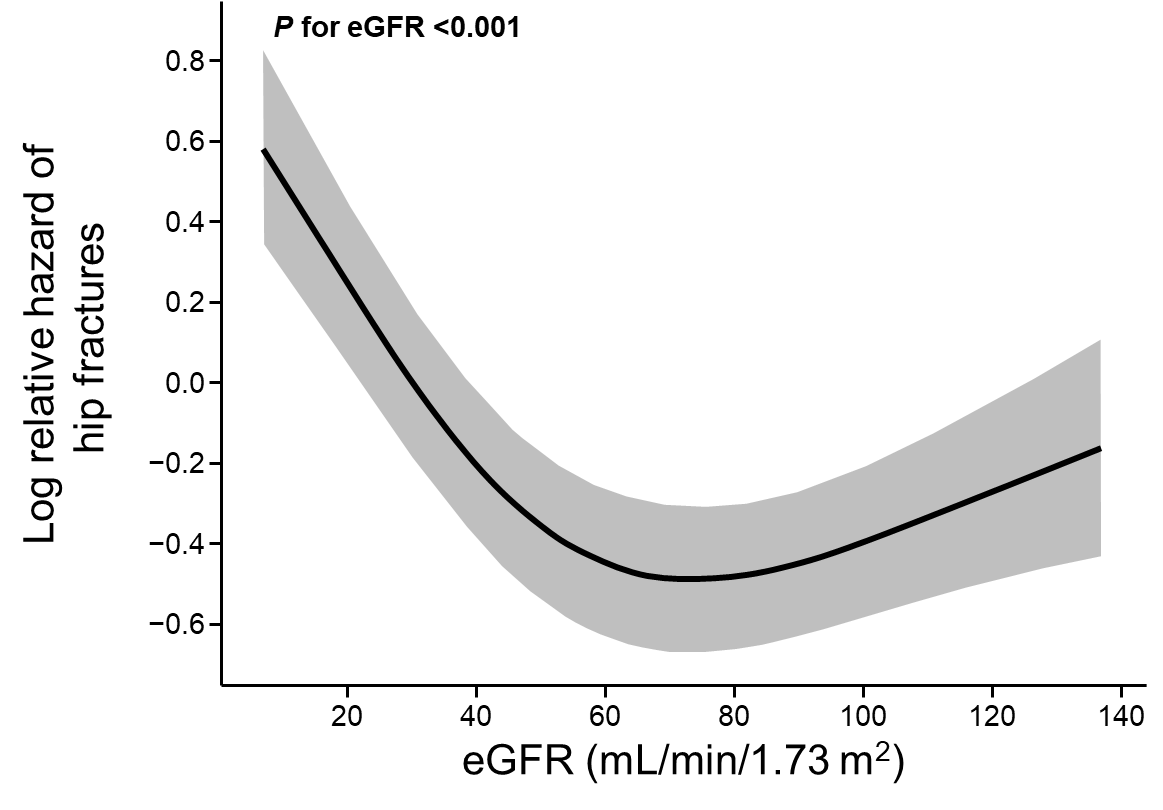
*

*eGFR* estimated glomerular filtration rate

**Supplementary Tables**

**Supplementary Table 1:** The ICD-10 codes used as diagnosis of fractures.

| **ICD-10 code** | **ICD-10 name** | **Disease code** | **Disease name** |
| --- | --- | --- | --- |
| **Hip, non-vertebral** | |  |  |
| S7200 | Fracture of neck of femur; closed | 8208003 | hip posterior fracture dislocation |
| S7200 | Fracture of neck of femur; closed | 8208004 | hip fracture |
| S7200 | Fracture of neck of femur; closed | 8208009 | femoral neck fracture |
| S7200 | Fracture of neck of femur; closed | 8208015 | hip central fracture dislocation |
| S7200 | Fracture of neck of femur; closed | 8833731 | hip fracture dislocation |
| S7200 | Fracture of neck of femur; closed | 8837297 | femoral neck transverse fracture |
| S7200 | Fracture of neck of femur; closed | 8837298 | pertrochanteric fracture |
| S7200 | Fracture of neck of femur; closed | 8837299 | intertrochanteric femoral fracture |
| S7200 | Fracture of neck of femur; closed | 8837300 | basicervical fracture |
| S7200 | Fracture of neck of femur; closed | 8837303 | intracapsular femoral neck fracture |
| S7200 | Fracture of neck of femur; closed | 8837305 | femoral subcapital fracture |
| S7200 | Fracture of neck of femur; closed | 8837306 | medial femoral head fracture |
| S7200 | Fracture of neck of femur; closed | 8837311 | femoral side neck fracture |
| S7200 | Fracture of neck of femur; closed | 8847522 | femoral proximal epiphyseal injury |
| S7201 | Fracture of neck of femur; open | 8841896 | intracapsular femoral neck open fracture |
| S7201 | Fracture of neck of femur; open | 8841897 | hip posterior open fracture dislocation |
| S7201 | Fracture of neck of femur; open | 8841898 | femoral neck open fracture |
| S7201 | Fracture of neck of femur; open | 8841899 | hip central open fracture dislocation |
| S7201 | Fracture of neck of femur; open | 8841900 | femoral neck open transverse fracture |
| S7201 | Fracture of neck of femur; open | 8841903 | hip open fracture dislocation |
| S7201 | Fracture of neck of femur; open | 8841904 | intertrochanteric femoral open fracture |
| S7201 | Fracture of neck of femur; open | 8841905 | hip joint open fracture |
| S7201 | Fracture of neck of femur; open | 8841906 | femoral side neck open fracture |
| S7210 | Pertrochanteric fracture; closed | 8837314 | trochanteric femoral fracture |
| S7210 | Pertrochanteric fracture; closed | 8837382 | greater trochanter fracture |
| S7210 | Pertrochanteric fracture; closed | 8837384 | greater trochanter avulsion fracture |
| S7210 | Pertrochanteric fracture; closed | 8837944 | intertrochanteric fracture |
| S7210 | Pertrochanteric fracture; closed | 8837945 | pertrochanteric fracture |
| S7211 | Pertrochanteric fracture; open | 8841910 | greater trochanter open fracture |
| S7211 | Pertrochanteric fracture; open | 8841911 | trochanteric femoral open fracture |
| S7220 | Subtrochanteric fracture; closed | 8837943 | subtrochanteric fracture |
| S7221 | Subtrochanteric fracture; open | 8841913 | subtrochanteric open fracture |
| S7230 | Fracture of shaft of femur; closed | 8210008 | femur shaft fracture |
| S7230 | Fracture of shaft of femur; closed | 8846314 | femur shaft comminuted fracture |
| S7231 | Fracture of shaft of femur; open | 8841914 | shaft of femur open fracture |
| S7231 | Fracture of shaft of femur; open | 8846313 | femur shaft open comminuted fracture |
| S7240 | Fracture of lower end of femur; closed | 8212005 | femoral condylar fracture |
| S7240 | Fracture of lower end of femur; closed | 8837290 | distal end of femur fracture |
| S7240 | Fracture of lower end of femur; closed | 8837292 | supracondylar femur fracture |
| S7240 | Fracture of lower end of femur; closed | 8837293 | lateral femoral condyle fracture |
| S7240 | Fracture of lower end of femur; closed | 8837316 | femoral internal condyle fracture |
| S7240 | Fracture of lower end of femur; closed | 8842346 | femoral distal epiphyseal injury |
| S7241 | Fracture of lower end of femur; open | 8841915 | distal end of femur open fracture |
| S7241 | Fracture of lower end of femur; open | 8841916 | femoral internal condyle open fracture |
| S7241 | Fracture of lower end of femur; open | 8841917 | supracondylar femur open fracture |
| S7241 | Fracture of lower end of femur; open | 8841918 | femoral condylar open fracture |
| S7241 | Fracture of lower end of femur; open | 8841919 | lateral femoral condyle open fracture |
| S7270 | Multiple fractures of femur; closed | 8837312 | femoral multiple fracture |
| S7271 | Multiple fractures of femur; open | 8841920 | femur multiple open fractures |
| S7290 | Fracture of femur, part unspecified; closed | 8210002 | femoral fracture |
| S7290 | Fracture of femur, part unspecified; closed | 8837320 | femoral incomplete fracture |
| S7290 | Fracture of femur, part unspecified; closed | 8837321 | femoral comminuted fracture |
| S7291 | Fracture of femur, part unspecified; open | 8837291 | femoral open fracture |
| S7291 | Fracture of femur, part unspecified; open | 8841990 | femoral comminuted open fracture |
| **Vertebral** |  |  |  |
| M4849 | Fatigue fracture of vertebra; Site unspecified | 8836024 | vertebral stress fracture |
| S2200 | Fracture of thoracic vertebra; closed | 8052003 | thoracic vertebral fracture |
| S2200 | Fracture of thoracic vertebra; closed | 8832545 | thoracic vertebral compression fracture |
| S2200 | Fracture of thoracic vertebra; closed | 8832552 | thoracic vertebral fracture dislocation |
| S2200 | Fracture of thoracic vertebra; closed | 8832577 | chest transverse process fracture |
| S2200 | Fracture of thoracic vertebra; closed | 8832615 | chest vertebral body fracture |
| S2200 | Fracture of thoracic vertebra; closed | 8842484 | thoracic vertebral spinous process fracture |
| S2200 | Fracture of thoracic vertebra; closed | 8842568 | thoracic vertebral bursting fracture |
| S2200 | Fracture of thoracic vertebra; closed | 8844853 | thoracic vertebral transverse process fracture |
| S2200 | Fracture of thoracic vertebra; closed | 8844856 | thoracic vertebral arch fracture |
| S2200 | Fracture of thoracic vertebra; closed | 8844858 | thoracic vertebral body fracture |
| S2200 | Fracture of thoracic vertebra; closed | 8848866 | thoracic vertebral occult fracture |
| S2201 | Fracture of thoracic vertebra; open | 8841766 | thoracic vertebral open fracture dislocation |
| S2201 | Fracture of thoracic vertebra; open | 8841768 | thoracic vertebral open fracture |
| S2201 | Fracture of thoracic vertebra; open | 8844852 | thoracic vertebral transverse process open fracture |
| S2201 | Fracture of thoracic vertebra; open | 8844854 | thoracic vertebral arch open fracture |
| S2201 | Fracture of thoracic vertebra; open | 8844855 | thoracic vertebral spinous process open fracture |
| S2201 | Fracture of thoracic vertebra; open | 8844857 | thoracic vertebral body open fracture |
| S2210 | Multiple fractures of thoracic spine; closed | 8837010 | multiple thoracic vertebral fracture |
| S2210 | Multiple fractures of thoracic spine; closed | 8845535 | thoracic vertebral multiple fracture |
| S2210 | Multiple fractures of thoracic spine; closed | 8845886 | thoracic vertebral multiple compression fracture |
| S2211 | Multiple fractures of thoracic spine; open | 8841770 | multiple thoracic vertebral open fracture |
| S2220 | Fracture of sternum; closed | 8072002 | sternal fracture |
| S2220 | Fracture of sternum; closed | 8832491 | sternal manubrium fracture |
| S2220 | Fracture of sternum; closed | 8832493 | fissure fracture of sternum |
| S2220 | Fracture of sternum; closed | 8832500 | sternal incomplete fracture |
| S2221 | Fracture of sternum; open | 8841771 | open fracture of sternum |
| S320 | Fracture of lumbar vertebra | 8064002 | lumbar vertebra injury |
| S3200 | Fracture of lumbar vertebra; closed | 8054003 | lumbar vertebral compression fracture |
| S3200 | Fracture of lumbar vertebra; closed | 8054007 | lumbar vertebra fracture dislocation |
| S3200 | Fracture of lumbar vertebra; closed | 8054016 | lumbar vertebral fracture |
| S3200 | Fracture of lumbar vertebra; closed | 8840814 | lumbar vertebral transverse process fracture |
| S3200 | Fracture of lumbar vertebra; closed | 8840815 | lumbar vertebral spinous process fracture |
| S3200 | Fracture of lumbar vertebra; closed | 8840825 | lumbar vertebral incomplete fracture |
| S3200 | Fracture of lumbar vertebra; closed | 8842635 | lumbar vertebra bursting fracture |
| S3200 | Fracture of lumbar vertebra; closed | 8844924 | lumbar vertebral arch fracture |
| S3200 | Fracture of lumbar vertebra; closed | 8844926 | lumbar vertebral body fracture |
| S3200 | Fracture of lumbar vertebra; closed | 8849001 | lumbar vertebral occult fracture |
| S3201 | Fracture of lumbar vertebra; open | 8841776 | lumbar vertebral open fracture |
| S3201 | Fracture of lumbar vertebra; open | 8841781 | lumbar vertebral transverse process open fracture |
| S3201 | Fracture of lumbar vertebra; open | 8844925 | lumbar vertebral body open fracture |
| S3270 | Multiple fractures of lumbar spine and pelvis; closed | 8835306 | double vertical pelvic fracture |
| S3270 | Multiple fractures of lumbar spine and pelvis; closed | 8840288 | Malgaigne fracture |
| S3270 | Multiple fractures of lumbar spine and pelvis; closed | 8845558 | pelvic multiple fracture |
| S3270 | Multiple fractures of lumbar spine and pelvis; closed | 8845616 | lumbar vertebral and pevis multiple fracture |
| S3270 | Multiple fractures of lumbar spine and pelvis; closed | 8845617 | lumbar vertebral multiple compression fracture |
| S3270 | Multiple fractures of lumbar spine and pelvis; closed | 8845618 | lumbar vertebral multiple fracture |
| S3271 | Multiple fractures of lumbar spine and pelvis; open | 8841788 | double vertical open fracture of pelvis |
| S3280 | Fracture of other and unspecified parts of lumbar spine and pelvis; closed | 8088001 | pelvic fracture |
| S3280 | Fracture of other and unspecified parts of lumbar spine and pelvis; closed | 8834126 | ischial tuberosity avulsion fracture |
| S3280 | Fracture of other and unspecified parts of lumbar spine and pelvis; closed | 8834129 | ischium fracture |
| S3280 | Fracture of other and unspecified parts of lumbar spine and pelvis; closed | 8840803 | lumbosacral spine fracture |
| S3280 | Fracture of other and unspecified parts of lumbar spine and pelvis; closed | 8847754 | ring fracture |
| S3281 | Fracture of other and unspecified parts of lumbar spine and pelvis; open | 8841789 | pelvis open fracture |
| S3281 | Fracture of other and unspecified parts of lumbar spine and pelvis; open | 8841790 | lumbosacral spine open fracture |
| S3281 | Fracture of other and unspecified parts of lumbar spine and pelvis; open | 8841791 | ischium open fracture |
| S3281 | Fracture of other and unspecified parts of lumbar spine and pelvis; open | 8847753 | open ring fracture |
| **Non-vertebral** | |  |  |
| S2230 | Fracture of rib; closed | 8070006 | rib fracture |
| S2230 | Fracture of rib; closed | 8070010 | rib incomplete fracture |
| S2230 | Fracture of rib; closed | 8070011 | costal cartilage fracture |
| S2230 | Fracture of rib; closed | 8849009 | rib occult fracture |
| S2231 | Fracture of rib; open | 8070012 | rib open fracture |
| S2240 | Multiple fractures of ribs; closed | 8070005 | rib fissure fracture |
| S2240 | Multiple fractures of ribs; closed | 8070007 | costal multiple fracture |
| S2241 | Multiple fractures of ribs; open | 8841774 | rib multiple open fractures |
| S3210 | Fracture of sacrum; closed | 8067003 | sacrum bone fissure fracture |
| S3210 | Fracture of sacrum; closed | 8836095 | sacrum bone fracture |
| S3211 | Fracture of sacrum; open | 8841783 | sacrum bone open fracture |
| S3220 | Fracture of coccyx; closed | 8056002 | fractured coccyx |
| S3221 | Fracture of coccyx; open | 8841784 | coccygeal bone open fracture |
| S3230 | Fracture of ilium; closed | 8837720 | ilium fracture |
| S3230 | Fracture of ilium; closed | 8837725 | ilium avulsion fracture |
| S3230 | Fracture of ilium; closed | 8846244 | anterior inferior iliac spine avulsion fracture |
| S3230 | Fracture of ilium; closed | 8846300 | anterior superior iliac spine avulsion fracture |
| S3231 | Fracture of ilium; open | 8841785 | ilium open fracture |
| S3240 | Fracture of acetabulum; closed | 8831514 | acetabulum fracture |
| S3241 | Fracture of acetabulum; open | 8841786 | acetabulum open fracture |
| S3250 | Fracture of pubis; closed | 8837470 | pubis fracture |
| S3251 | Fracture of pubis; open | 8841787 | pubic bone open fracture |
| S4200 | Fracture of clavicle; closed | 8100003 | clavicle fracture |
| S4200 | Fracture of clavicle; closed | 8833996 | distal end of clavicle fracture |
| S4200 | Fracture of clavicle; closed | 8834002 | clavicle fissure fracture |
| S4200 | Fracture of clavicle; closed | 8834003 | clavicle acromion fracture |
| S4200 | Fracture of clavicle; closed | 8834005 | clavicular shaft fracture |
| S4200 | Fracture of clavicle; closed | 8848714 | clavicular comminuted fracture |
| S4201 | Fracture of clavicle; open | 8841792 | clavicle open fracture |
| S4201 | Fracture of clavicle; open | 8841794 | acromial end of clavicle open fracture |
| S4201 | Fracture of clavicle; open | 8841795 | shaft of clavicle open fracture |
| S4201 | Fracture of clavicle; open | 8841796 | distal end of clavicle open fracture |
| S4210 | Fracture of scapula; closed | 8110001 | shoulder fracture dislocation |
| S4210 | Fracture of scapula; closed | 8110003 | scapula acromion fracture |
| S4210 | Fracture of scapula; closed | 8110004 | scapula fracture |
| S4210 | Fracture of scapula; closed | 8833225 | scapula coracoid process fracture |
| S4210 | Fracture of scapula; closed | 8833226 | scapula glenoid fracture |
| S4210 | Fracture of scapula; closed | 8833228 | scapular neck fracture |
| S4210 | Fracture of scapula; closed | 8833229 | scapula body fracture |
| S4211 | Fracture of scapula; open | 8841798 | shoulder open fracture dislocation |
| S4211 | Fracture of scapula; open | 8841799 | scapula coracoid process open fracture |
| S4211 | Fracture of scapula; open | 8841801 | scapula open fracture |
| S4211 | Fracture of scapula; open | 8841802 | scapular glenoid open fracture |
| S4211 | Fracture of scapula; open | 8841803 | acromial process of scapula open fracture |
| S4220 | Fracture of upper end of humerus; closed | 8120003 | humeral surgical neck fracture |
| S4220 | Fracture of upper end of humerus; closed | 8120006 | humeral head fracture |
| S4220 | Fracture of upper end of humerus; closed | 8122002 | humeral neck fracture |
| S4220 | Fracture of upper end of humerus; closed | 8835500 | humeral anatomical neck fracture |
| S4220 | Fracture of upper end of humerus; closed | 8835505 | proximal end of humerus fracture |
| S4220 | Fracture of upper end of humerus; closed | 8835506 | proximal end of humerus comminuted fracture |
| S4220 | Fracture of upper end of humerus; closed | 8835510 | humeral surgical neck fracture dislocation |
| S4220 | Fracture of upper end of humerus; closed | 8835517 | tuberculum majus humeri fracture |
| S4220 | Fracture of upper end of humerus; closed | 8835518 | tuberculum majus humeri avulsion fracture |
| S4220 | Fracture of upper end of humerus; closed | 8847369 | humeral proximal epiphyseal injury |
| S4220 | Fracture of upper end of humerus; closed | 8847498 | humeral nodular fracture |
| S4221 | Fracture of upper end of humerus; open | 8841804 | humeral neck open fracture |
| S4221 | Fracture of upper end of humerus; open | 8841805 | proximal end of humerus open comminuted fracture |
| S4221 | Fracture of upper end of humerus; open | 8841806 | humeral head open fracture |
| S4221 | Fracture of upper end of humerus; open | 8841807 | proximal end of humerus open fracture |
| S4221 | Fracture of upper end of humerus; open | 8841811 | humeral surgical neck open fracture |
| S4230 | Fracture of shaft of humerus; closed | 8120005 | humeral fracture |
| S4230 | Fracture of shaft of humerus; closed | 8835512 | humeral shaft fracture |
| S4230 | Fracture of shaft of humerus; closed | 8835519 | humerus fracture dislocation |
| S4230 | Fracture of shaft of humerus; closed | 8835521 | humeral comminuted fracture |
| S4230 | Fracture of shaft of humerus; closed | 8847499 | humeral spiral fracture |
| S4231 | Fracture of shaft of humerus; open | 8841812 | humerus open fracture |
| S4231 | Fracture of shaft of humerus; open | 8841813 | humerus open comminuted fracture |
| S4231 | Fracture of shaft of humerus; open | 8841814 | humerus open fracture dislocation |
| S4231 | Fracture of shaft of humerus; open | 8841815 | humeral shaft open fracture |
| S4240 | Fracture of lower end of humerus; closed | 8124003 | lateral humeral condylar fracture |
| S4240 | Fracture of lower end of humerus; closed | 8124004 | medial humeral epicondyle fracture |
| S4240 | Fracture of lower end of humerus; closed | 8124005 | humeral medial condyle fracture |
| S4240 | Fracture of lower end of humerus; closed | 8124006 | supracondylar humeral fracture |
| S4240 | Fracture of lower end of humerus; closed | 8124007 | humeral condylar fracture |
| S4240 | Fracture of lower end of humerus; closed | 8124009 | lateral humeral epicondylar fracture |
| S4240 | Fracture of lower end of humerus; closed | 8835498 | distal end of humerus fracture |
| S4240 | Fracture of lower end of humerus; closed | 8835499 | distal end of humerus comminuted fracture |
| S4240 | Fracture of lower end of humerus; closed | 8835501 | humeral intercondylar fracture |
| S4240 | Fracture of lower end of humerus; closed | 8835502 | humeral condylar comminuted fracture |
| S4240 | Fracture of lower end of humerus; closed | 8835514 | capitulum humeri fracture |
| S4240 | Fracture of lower end of humerus; closed | 8842343 | humeral distal epiphyseal injury |
| S4240 | Fracture of lower end of humerus; closed | 8842344 | humeral transcondylar fracture |
| S4240 | Fracture of lower end of humerus; closed | 8847495 | humeral trochlea fracture |
| S4241 | Fracture of lower end of humerus; open | 8841817 | medial humeral epicondyle open fracture |
| S4241 | Fracture of lower end of humerus; open | 8841818 | distal end of humerus open comminuted fracture |
| S4241 | Fracture of lower end of humerus; open | 8841819 | humeral condylar open comminuted fracture |
| S4241 | Fracture of lower end of humerus; open | 8841820 | humeral medial condyle open fracture |
| S4241 | Fracture of lower end of humerus; open | 8841821 | supracondylar humeral open fracture |
| S4241 | Fracture of lower end of humerus; open | 8841822 | capitulum humeri open fracture |
| S4241 | Fracture of lower end of humerus; open | 8841823 | lateral humeral condylar open fracture |
| S4241 | Fracture of lower end of humerus; open | 8841824 | distal end of humerus open fracture |
| S4241 | Fracture of lower end of humerus; open | 8841825 | lateral humeral epicondylar open fracture |
| S4241 | Fracture of lower end of humerus; open | 8841826 | humeral condylar open fracture |
| S4241 | Fracture of lower end of humerus; open | 8845330 | humeral transcondylar open fracture |
| S4290 | Fracture of shoulder girdle, part unspecified; closed | 8831345 | shoulder fracture |
| S4291 | Fracture of shoulder girdle, part unspecified; open | 8841828 | shoulder open fracture |
| S5200 | Fracture of upper end of ulna; closed | 8124012 | elbow fracture |
| S5200 | Fracture of upper end of ulna; closed | 8124013 | elbow fracture dislocation |
| S5200 | Fracture of upper end of ulna; closed | 8124015 | olecranon fracture |
| S5200 | Fracture of upper end of ulna; closed | 8130007 | ulnar olecranon fracture |
| S5200 | Fracture of upper end of ulna; closed | 8834510 | proximal end of ulna fracture |
| S5200 | Fracture of upper end of ulna; closed | 8834513 | ulnar coronoid process fracture |
| S5200 | Fracture of upper end of ulna; closed | 8840685 | Monteggia’s fracture |
| S5200 | Fracture of upper end of ulna; closed | 8848084 | intra-articular elbow fracture |
| S5200 | Fracture of upper end of ulna; closed | 8848747 | olecranon comminuted fracture |
| S5200 | Fracture of upper end of ulna; closed | 8849636 | ulna proximal epiphyseal injury |
| S5201 | Fracture of upper end of ulna; open | 8841829 | proximal end of ulna open fracture |
| S5201 | Fracture of upper end of ulna; open | 8841830 | ulnar olecranon open fracture |
| S5201 | Fracture of upper end of ulna; open | 8841831 | elbow open fracture dislocation |
| S5201 | Fracture of upper end of ulna; open | 8841832 | coronoid process of ulna open fracture |
| S5201 | Fracture of upper end of ulna; open | 8841833 | elbow open fracture |
| S5210 | Fracture of upper end of radius; closed | 8838019 | proximal end of radius fracture |
| S5210 | Fracture of upper end of radius; closed | 8838022 | radius neck fracture |
| S5210 | Fracture of upper end of radius; closed | 8838032 | radial head fracture |
| S5210 | Fracture of upper end of radius; closed | 8845139 | radial head comminuted fracture |
| S5211 | Fracture of upper end of radius; open | 8841834 | head of radius open fracture |
| S5211 | Fracture of upper end of radius; open | 8841835 | radius neck open fracture |
| S5211 | Fracture of upper end of radius; open | 8841836 | proximal end of radius open fracture |
| S5220 | Fracture of shaft of ulna; closed | 8130005 | ulna fracture |
| S5220 | Fracture of shaft of ulna; closed | 8132001 | ulnar shaft fracture |
| S5220 | Fracture of shaft of ulna; closed | 8834509 | ulna fissure fracture |
| S5221 | Fracture of shaft of ulna; open | 8841837 | shaft of ulna open fracture |
| S5221 | Fracture of shaft of ulna; open | 8841838 | ulna open fracture |
| S5230 | Fracture of shaft of radius; closed | 8132004 | radius shaft fracture |
| S5230 | Fracture of shaft of radius; closed | 8838018 | radius fissure fracture |
| S5231 | Fracture of shaft of radius; open | 8841839 | shaft of radius open fracture |
| S5240 | Fracture of shafts of both ulna and radius; closed | 8838026 | radioulnar fracture |
| S5240 | Fracture of shafts of both ulna and radius; closed | 8845138 | radioulnar shaft fracture |
| S5241 | Fracture of shafts of both ulna and radius; open | 8841848 | radioulnar open fracture |
| S5241 | Fracture of shafts of both ulna and radius; open | 8845137 | radioulnar shaft open fracture |
| S5250 | Fracture of lower end of radius; closed | 8134002 | Smith’s fracture |
| S5250 | Fracture of lower end of radius; closed | 8134017 | Galeazzi’s fracture |
| S5250 | Fracture of lower end of radius; closed | 8134018 | Barton’s fracture |
| S5250 | Fracture of lower end of radius; closed | 8833324 | Colles’ fracture |
| S5250 | Fracture of lower end of radius; closed | 8838017 | distal end of radius fracture |
| S5250 | Fracture of lower end of radius; closed | 8838021 | radius styloid process fracture |
| S5250 | Fracture of lower end of radius; closed | 8842348 | distal end of radius epiphyseal injury |
| S5250 | Fracture of lower end of radius; closed | 8845136 | distal end of radius comminuted fracture |
| S5250 | Fracture of lower end of radius; closed | 8848088 | intra-articular fracture of distal end of radius |
| S5251 | Fracture of lower end of radius; open | 8841840 | distal end of radius open fracture |
| S5251 | Fracture of lower end of radius; open | 8841841 | radius styloid process open fracture |
| S5251 | Fracture of lower end of radius; open | 8845135 | distal end of radius open comminuted fracture |
| S5260 | Fracture of lower end of both ulna and radius; closed | 8838025 | distal radioulnar fracture |
| S5260 | Fracture of lower end of both ulna and radius; closed | 8848984 | distal end of radioulnar fracture |
| S5261 | Fracture of lower end of both ulna and radius; open | 8841842 | distal radioulnar open fracture |
| S5261 | Fracture of lower end of both ulna and radius; open | 8848846 | distal end of radioulnar open fracture |
| S5270 | Multiple fractures of forearm; closed | 8836610 | forearm multiple fracture |
| S5271 | Multiple fractures of forearm; open | 8841843 | forearm multiple open fractures |
| S5280 | Fracture of other parts of forearm; closed | 8130016 | radius fracture |
| S5280 | Fracture of other parts of forearm; closed | 8834506 | distal end of ulna fracture |
| S5280 | Fracture of other parts of forearm; closed | 8834511 | ulnar styloid process fracture |
| S5280 | Fracture of other parts of forearm; closed | 8834516 | ulnar head fracture |
| S5281 | Fracture of other parts of forearm; open | 8841844 | head of ulna open fracture |
| S5281 | Fracture of other parts of forearm; open | 8841845 | radius open fracture |
| S5281 | Fracture of other parts of forearm; open | 8841846 | distal end of ulna open fracture |
| S5281 | Fracture of other parts of forearm; open | 8841847 | ulnar styloid process open fracture |
| S5290 | Fracture of forearm, part unspecified; closed | 8836598 | forearm fracture |
| S5290 | Fracture of forearm, part unspecified; closed | 8836628 | forearm greenstick fracture |
| S5291 | Fracture of forearm, part unspecified; open | 8831043 | open forearm fracture |
| S8200 | Fracture of patella; closed | 8220003 | patella fracture |
| S8200 | Fracture of patella; closed | 8220006 | knee joint fracture |
| S8200 | Fracture of patella; closed | 8220007 | knee joint fracture dislocation |
| S8200 | Fracture of patella; closed | 8220008 | intra-articular knee fracture |
| S8200 | Fracture of patella; closed | 8834425 | patella incomplete fracture |
| S8200 | Fracture of patella; closed | 8834426 | patella comminuted fracture |
| S8200 | Fracture of patella; closed | 8848894 | patellar occult fracture |
| S8201 | Fracture of patella; open | 8841922 | knee joint open fracture dislocation |
| S8201 | Fracture of patella; open | 8841923 | patellar open comminuted fracture |
| S8201 | Fracture of patella; open | 8841924 | knee joint open fracture |
| S8201 | Fracture of patella; open | 8841991 | patellar open fracture |
| S8210 | Fracture of upper end of tibia; closed | 8230036 | tibial plateau fracture |
| S8210 | Fracture of upper end of tibia; closed | 8832890 | tibial intercondylar eminence fracture |
| S8210 | Fracture of upper end of tibia; closed | 8832891 | tibial condylar fracture |
| S8210 | Fracture of upper end of tibia; closed | 8832892 | tibial condylar avulsion fracture |
| S8210 | Fracture of upper end of tibia; closed | 8832893 | tibial condylar comminuted fracture |
| S8210 | Fracture of upper end of tibia; closed | 8832895 | lateral tibial condyle avulsion fracture |
| S8210 | Fracture of upper end of tibia; closed | 8832898 | proximal end of tibia fracture |
| S8210 | Fracture of upper end of tibia; closed | 8832899 | proximal end of tibia comminuted fracture |
| S8210 | Fracture of upper end of tibia; closed | 8832904 | tibial tubercle avulsion fracture |
| S8210 | Fracture of upper end of tibia; closed | 8832906 | intraarticular fracture of the knee |
| S8210 | Fracture of upper end of tibia; closed | 8832909 | tibial tuberosity fracture |
| S8210 | Fracture of upper end of tibia; closed | 8843712 | posterior cruciate ligament attachment avulsion fracture |
| S8210 | Fracture of upper end of tibia; closed | 8843745 | anterior cruciate ligament attachment avulsion fracture |
| S8210 | Fracture of upper end of tibia; closed | 8846262 | proximal end of tibia and fibula fracture |
| S8210 | Fracture of upper end of tibia; closed | 8846263 | proximal end of tibia and fibula comminuted fracture |
| S8210 | Fracture of upper end of tibia; closed | 8847464 | tibia proximal epiphyseal injury |
| S8210 | Fracture of upper end of tibia; closed | 8848869 | proximal end of tibia occult fracture |
| S8211 | Fracture of upper end of tibia; open | 8841925 | tibial tuberosity open fracture |
| S8211 | Fracture of upper end of tibia; open | 8841926 | tibial condylar open comminuted fracture |
| S8211 | Fracture of upper end of tibia; open | 8841927 | tibial plateau open fracture |
| S8211 | Fracture of upper end of tibia; open | 8841928 | tibial condylar open fracture |
| S8211 | Fracture of upper end of tibia; open | 8841929 | proximal end of tibia open fracture |
| S8211 | Fracture of upper end of tibia; open | 8841930 | proximal end of tibia open comminuted fracture |
| S8211 | Fracture of upper end of tibia; open | 8841931 | tibial intercondylar eminence open fracture |
| S8211 | Fracture of upper end of tibia; open | 8846260 | proximal end of tibia and fibula open fracture |
| S8211 | Fracture of upper end of tibia; open | 8846261 | proximal end of tibia and fibula open comminuted fracture |
| S8220 | Fracture of shaft of tibia; closed | 8230014 | tibial fracture |
| S8220 | Fracture of shaft of tibia; closed | 8232005 | shaft of tibia fracture |
| S8220 | Fracture of shaft of tibia; closed | 8832911 | tibial comminuted fracture |
| S8220 | Fracture of shaft of tibia; closed | 8839229 | tibial and fibula fracture |
| S8220 | Fracture of shaft of tibia; closed | 8846266 | tibial and fibula shaft fracture |
| S8220 | Fracture of shaft of tibia; closed | 8846267 | tibial and fibula shaft comminuted fracture |
| S8220 | Fracture of shaft of tibia; closed | 8848870 | tibial occult fracture |
| S8221 | Fracture of shaft of tibia; open | 8841932 | tibial and fibula open fracture |
| S8221 | Fracture of shaft of tibia; open | 8841933 | shaft of tibia open fracture |
| S8221 | Fracture of shaft of tibia; open | 8841934 | tibial open comminuted fracture |
| S8221 | Fracture of shaft of tibia; open | 8841992 | tibia open fracture |
| S8221 | Fracture of shaft of tibia; open | 8846264 | tibial and fibula shaft open fracture |
| S8221 | Fracture of shaft of tibia; open | 8846265 | tibial and fibula shaft open comminuted fracture |
| S8230 | Fracture of lower end of tibia; closed | 8832889 | distal end oftibia fracture |
| S8230 | Fracture of lower end of tibia; closed | 8842342 | tibial distal epiphyseal injury |
| S8230 | Fracture of lower end of tibia; closed | 8844041 | plafond fracture |
| S8230 | Fracture of lower end of tibia; closed | 8846258 | distal end of tibia and fibula fracture |
| S8230 | Fracture of lower end of tibia; closed | 8846259 | distal end of tibia and fibula comminuted fracture |
| S8231 | Fracture of lower end of tibia; open | 8841935 | distal end of tibia open fracture |
| S8231 | Fracture of lower end of tibia; open | 8844040 | plafond open fracture |
| S8231 | Fracture of lower end of tibia; open | 8846256 | distal end of tibia and fibula open fracture |
| S8231 | Fracture of lower end of tibia; open | 8846257 | distal end of tibia and fibula open comminuted fracture |
| S8240 | Fracture of fibula alone; closed | 8230026 | fibula fissure fracture |
| S8240 | Fracture of fibula alone; closed | 8230027 | fracture of fibula |
| S8240 | Fracture of fibula alone; closed | 8230030 | fibula avulsion fracture |
| S8240 | Fracture of fibula alone; closed | 8232006 | shaft of fibula fracture |
| S8240 | Fracture of fibula alone; closed | 8839223 | fibular head fracture |
| S8240 | Fracture of fibula alone; closed | 8839224 | fibular head comminuted fracture |
| S8240 | Fracture of fibula alone; closed | 8839225 | distal end of fibula fracture |
| S8240 | Fracture of fibula alone; closed | 8839226 | distal end of fibula avulsion fracture |
| S8240 | Fracture of fibula alone; closed | 8839228 | proximal end of fibula fracture |
| S8240 | Fracture of fibula alone; closed | 8847550 | fibula proximal epiphyseal injury |
| S8241 | Fracture of fibula alone; open | 8839227 | fibula open fracture |
| S8241 | Fracture of fibula alone; open | 8841752 | fibula open comminuted fracture |
| S8241 | Fracture of fibula alone; open | 8841936 | shaft of fibula open fracture |
| S8241 | Fracture of fibula alone; open | 8841938 | proximal end of fibula open fracture |
| S8241 | Fracture of fibula alone; open | 8841939 | head of fibula open fracture |
| S8241 | Fracture of fibula alone; open | 8841940 | distal end of fibula open fracture |
| S8250 | Fracture of medial malleolus; closed | 8842198 | medial malleolus fracture |
| S8250 | Fracture of medial malleolus; closed | 8846312 | posterior malleolus fracture |
| S8251 | Fracture of medial malleolus; open | 8842197 | medial malleolus open fracture |
| S8251 | Fracture of medial malleolus; open | 8846311 | posterior malleolus open fracture |
| S8260 | Fracture of lateral malleolus; closed | 8244006 | Dupuytren’s fracture |
| S8260 | Fracture of lateral malleolus; closed | 8842193 | lateral malleolar fracture |
| S8260 | Fracture of lateral malleolus; closed | 8842195 | lateral malleolar avulsion fracture |
| S8260 | Fracture of lateral malleolus; closed | 8842351 | fibular distal epiphyseal injury |
| S8261 | Fracture of lateral malleolus; open | 8842192 | lateral malleolar open fracture |
| S8261 | Fracture of lateral malleolus; open | 8842194 | lateral malleolar avulsion open fracture |
| S8270 | Multiple fractures of lower leg; closed | 8831310 | lower leg multiple fracture |
| S8270 | Multiple fractures of lower leg; closed | 8832888 | distal proximal tibial fracture |
| S8271 | Multiple fractures of lower leg; open | 8841944 | distal proximal tibial open fracture |
| S8271 | Multiple fractures of lower leg; open | 8841945 | lower leg multiple open fractures |
| S8280 | Fractures of other parts of lower leg; closed | 8248001 | ankle fracture |
| S8280 | Fractures of other parts of lower leg; closed | 8248002 | talocrural articulation fracture dislocation |
| S8280 | Fractures of other parts of lower leg; closed | 8831438 | malleolar fracture |
| S8280 | Fractures of other parts of lower leg; closed | 8834066 | trimalleolar fracture |
| S8280 | Fractures of other parts of lower leg; closed | 8836711 | fracture of ankle anterior tubercle |
| S8280 | Fractures of other parts of lower leg; closed | 8836713 | talocrural articulation comminuted fracture dislocation |
| S8280 | Fractures of other parts of lower leg; closed | 8841011 | bilateral malleolar fractures |
| S8280 | Fractures of other parts of lower leg; closed | 8848081 | intra-articular ankle fracture |
| S8281 | Fractures of other parts of lower leg; open | 8841946 | talocrural articulation open fracture dislocation |
| S8281 | Fractures of other parts of lower leg; open | 8841948 | bilateral malleolar open fractures |
| S8281 | Fractures of other parts of lower leg; open | 8841949 | talocrural articulation open comminuted fracture dislocation |
| S8281 | Fractures of other parts of lower leg; open | 8841951 | malleolar open fracture |
| S8281 | Fractures of other parts of lower leg; open | 8841993 | talocrural articulation open fracture |
| S8281 | Fractures of other parts of lower leg; open | 8846992 | trimalleolar open fracture |
| S8281 | Fractures of other parts of lower leg; open | 8849522 | talocrural articulation open comminuted fracture dislocation |
| **All** |  |  |  |
| M8433 | Stress fracture, not elsewhere classified, Forearm | 8847024 | ulnar stress fracture |
| M8434 | Stress fracture, not elsewhere classified, Hand | 8848386 | thumb metacarpal bone stress fracture |
| M8434 | Stress fracture, not elsewhere classified, Hand | 8848393 | metacarpal bone stress fracture |
| M8435 | Stress fracture, not elsewhere classified, Pelvic region and thighs | 8834137 | ischiatic stress fracture |
| M8435 | Stress fracture, not elsewhere classified, Pelvic region and thighs | 8847047 | femoral neck stress fracture |
| M8435 | Stress fracture, not elsewhere classified, Pelvic region and thighs | 8847048 | femoral diaphyseal stress fracture |
| M8435 | Stress fracture, not elsewhere classified, Pelvic region and thighs | 8847049 | femoral stress fracture |
| M8435 | Stress fracture, not elsewhere classified, Pelvic region and thighs | 8847050 | pubic stress fracture |
| M8436 | Stress fracture, not elsewhere classified,Lower leg | 8230017 | tibial stress fracture |
| M8436 | Stress fracture, not elsewhere classified,Lower leg | 8847062 | fibular stress fracture |
| M8437 | Stress fracture, not elsewhere classified, Ankle and foot | 8252020 | march fracture |
| M8437 | Stress fracture, not elsewhere classified, Ankle and foot | 8847025 | navicular stress fracture |
| M8437 | Stress fracture, not elsewhere classified, Ankle and foot | 8847028 | calcaneal stress fracture |
| M8437 | Stress fracture, not elsewhere classified, Ankle and foot | 8847053 | metatarsal stress fracture |
| M8438 | Stress fracture, not elsewhere classified,Other | 8070009 | costal stress fracture |
| M8439 | Stress fracture, not elsewhere classified, Site unknown | 7303009 | fatigue periosteum failure |
| M8439 | Stress fracture, not elsewhere classified, Site unknown | 7331016 | stress fracture |
| T0210 | Fractures involving thorax with lower back and pelvic region | 8832654 | thoracolumbar spine compression fracture |
| T0220 | First cervical fracture; closed | 8830539 | unilateral upper limb multiple fracture |
| T0220 | First cervical fracture; closed | 8835543 | humerus and forearm fracture |
| T0220 | First cervical fracture; closed | 8838036 | distal radial carpal bone fracture |
| T0221 | First cervical fracture; open | 8841971 | unilateral upper extremity multiple open fractures |
| T0221 | First cervical fracture; open | 8841972 | humerus and forearm open fracture |
| T0230 | Multisite fracture of the lower extremity on one side; closed | 8830538 | unilateral leg multiple fracture |
| T0231 | Multisite fracture of the lower extremity on one side; open | 8841973 | unilateral leg multiple open fractures |
| T0240 | Multisite fractures of bilateral upper extremities; closed | 8841039 | both upper limbs multiple fracture |
| T0241 | Multisite fractures of bilateral upper extremities; open | 8841974 | bilateral upper extremity multiple open fractures |
| T0250 | Multisite fractures of bilateral lower extremities; closed | 8841038 | both legs multiple fracture |
| T0251 | Multisite fractures of bilateral lower extremities; open | 8841975 | both legs multiple open fractures |
| T0260 | Fractures of multiple sites of the upper extremity, with fractures of the lower extremity; closed | 8845564 | Multiple upper extremity fractures and lower extremity fracture complications |
| T0280 | Other compound site fractures; closed | 8849450 | combined site fracture |
| T0281 | Other compound site fractures; open | 8849449 | combined site open fracture |
| T0290 | Multiple fractures, unspecified, closed | 8290022 | multiple fracture |
| T0290 | Multiple fractures, unspecified, closed | 8831220 | leg multiple fracture |
| T0291 | Multiple fractures, unspecified, open | 8290023 | multiple complex fracture |
| T0291 | Multiple fractures, unspecified, open | 8841976 | multiple open fractures |
| T0291 | Multiple fractures, unspecified, open | 8841977 | leg multiple open fractures |
| T08-0 | Fracture of spine, level unspecified, closed | 8050021 | Chance fracture |
| T08-0 | Fracture of spine, level unspecified, closed | 8050022 | seat belt fracture |
| T08-0 | Fracture of spine, level unspecified, closed | 8058002 | spine compression fracture |
| T08-0 | Fracture of spine, level unspecified, closed | 8058003 | spinal fracture |
| T08-0 | Fracture of spine, level unspecified, closed | 8058005 | vertebral arch fracture |
| T08-0 | Fracture of spine, level unspecified, closed | 8058006 | vertebral body compression fracture |
| T08-0 | Fracture of spine, level unspecified, closed | 8058007 | vertebral body fracture |
| T08-0 | Fracture of spine, level unspecified, closed | 8058008 | vertebral body fracture dislocation |
| T08-0 | Fracture of spine, level unspecified, closed | 8058011 | posterior element fracture |
| T08-0 | Fracture of spine, level unspecified, closed | 8830972 | transverse process fracture |
| T08-0 | Fracture of spine, level unspecified, closed | 8832673 | spinous process fracture |
| T08-0 | Fracture of spine, level unspecified, closed | 8837833 | vertebral angle transection |
| T08-1 | Fracture of spine, level unspecified, open | 8841979 | transverse process open fracture |
| T10-0 | Fracture of upper limb, level unspecified, closed | 8180001 | upper limb fracture |
| T10-1 | Fracture of upper limb, level unspecified, open | 8841985 | upper limb open fracture |
| T1420 | Fracture of unspecified body region, closed | 8022008 | greenstick fracture |
| T1420 | Fracture of unspecified body region, closed | 8029001 | transverse fracture |
| T1420 | Fracture of unspecified body region, closed | 8029004 | longitudinal fracture |
| T1420 | Fracture of unspecified body region, closed | 8029005 | double fracture |
| T1420 | Fracture of unspecified body region, closed | 8029007 | bursting fracture |
| T1420 | Fracture of unspecified body region, closed | 8290002 | complete fracture |
| T1420 | Fracture of unspecified body region, closed | 8290003 | articular fracture |
| T1420 | Fracture of unspecified body region, closed | 8290004 | joint fracture dislocation |
| T1420 | Fracture of unspecified body region, closed | 8290011 | fissure fracture |
| T1420 | Fracture of unspecified body region, closed | 8290015 | fracture |
| T1420 | Fracture of unspecified body region, closed | 8290019 | sesamoid bone fracture |
| T1420 | Fracture of unspecified body region, closed | 8290021 | linear fracture |
| T1420 | Fracture of unspecified body region, closed | 8290024 | fracture dislocation |
| T1420 | Fracture of unspecified body region, closed | 8290025 | simple fracture |
| T1420 | Fracture of unspecified body region, closed | 8290029 | avulsion fracture |
| T1420 | Fracture of unspecified body region, closed | 8290035 | incomplete fracture |
| T1420 | Fracture of unspecified body region, closed | 8290038 | avulsion fracture |
| T1420 | Fracture of unspecified body region, closed | 8290043 | spiral fracture |
| T1420 | Fracture of unspecified body region, closed | 8290045 | closed fracture |
| T1420 | Fracture of unspecified body region, closed | 8290047 | flexion fracture |
| T1420 | Fracture of unspecified body region, closed | 8290048 | compression fracture |
| T1420 | Fracture of unspecified body region, closed | 8831676 | depressed fracture |
| T1420 | Fracture of unspecified body region, closed | 8834503 | oblique fracture |
| T1420 | Fracture of unspecified body region, closed | 8837927 | displaced fracture |
| T1420 | Fracture of unspecified body region, closed | 8839812 | comminuted fracture |
| T1420 | Fracture of unspecified body region, closed | 8840958 | iastatic fracture |
| T1420 | Fracture of unspecified body region, closed | 8848035 | intra-articular fracture |
| T1420 | Fracture of unspecified body region, closed | 8848996 | occult fracture |
| T1421 | Fracture of unspecified body region, open | 8290036 | complex fracture |
| T1421 | Fracture of unspecified body region, open | 8291005 | open fracture |
| T1421 | Fracture of unspecified body region, open | 8291006 | open dislocation fracture |
| T1421 | Fracture of unspecified body region, open | 8291007 | open comminuted fracture |
| T1421 | Fracture of unspecified body region, open | 8841986 | open depressed fracture |

**Supplementary Table 2** Incidence of vertebral and non-vertebral fracture.

|  |  | ***n*** | **Vertebral fracture** | **Non-vertebral fracture** |
| --- | --- | --- | --- | --- |
| Overall |  | 76,598 | 1,528 (2.0) | 3,023 (3.9) |
| Group | Control | 38,299 | 790 (2.1) | 1,497 (3.9) |
|  | CKD | 38,299 | 738 (1.9) | 1,526 (4.0) |
| CKD stage | G3a | 10,111 | 132 (1.3) | 270 (2.7) |
|  | G3b | 12,763 | 259 (2.0) | 450 (3.5) |
|  | G4–5 | 15,425 | 347 (2.2) | 806 (5.2) |
| Age group (years) | 10s | 24 | 0 (0.0) | 0 (0.0) |
|  | 20s | 208 | 1 (0.5) | 0 (0.0) |
|  | 30s | 1,034 | 2 (0.2) | 14 (1.4) |
|  | 40s | 3,554 | 11 (0.3) | 77 (2.2) |
|  | 50s | 7,114 | 41 (0.6) | 164 (2.3) |
|  | 60s | 14,928 | 171 (1.1) | 467 (3.1) |
|  | 70s | 24,870 | 569 (2.3) | 963 (3.9) |
|  | 80s | 21,302 | 652 (3.1) | 1,135 (5.3) |
|  | 90s | 3,514 | 80 (2.3) | 202 (5.7) |
|  | ≥100 | 50 | 1 (2.0) | 1 (2.0) |
| Sex | Male | 51,342 | 796 (1.6) | 1,498 (2.9) |
|  | Female | 25,256 | 732 (2.9) | 1,525 (6.0) |
| Complications | Hypertension | 46,034 | 995 (2.2) | 1,957 (4.3) |
|  | Dyslipidemia | 31,624 | 609 (1.9) | 1,251 (4.0) |
|  | Hyperuricemia | 16,242 | 292 (1.8) | 578 (3.6) |
|  | Diabetes | 32,714 | 615 (1.9) | 1,352 (4.1) |
|  | Alcoholism | 190 | 4 (2.1) | 7 (3.7) |
|  | Rheumatoid arthritis | 2,596 | 77 (3.0) | 124 (4.8) |
|  | Dementia | 4,090 | 115 (2.8) | 244 (6.0) |
|  | Sleeping disorder | 12,783 | 370 (2.9) | 677 (5.3) |
|  | COPD | 2,946 | 81 (2.7) | 116 (3.9) |
| Drugs | Glucocorticoid | 3,673 | 115 (3.1) | 165 (4.5) |
|  | Proton pump inhibitor | 25,176 | 579 (2.3) | 1,104 (4.4) |
|  | Hormonal therapy | 132 | 4 (3.0) | 6 (4.5) |
|  | Thiazoline | 1,665 | 41 (2.5) | 110 (6.6) |
|  | Beta-blocker | 14,837 | 262 (1.8) | 539 (3.6) |
|  | Loop diuretics | 14,762 | 401 (2.7) | 777 (5.3) |
|  | Heparin | 9,907 | 183 (1.8) | 343 (3.5) |
|  | Warfarin | 4,489 | 171 (3.8) | 245 (5.5) |
|  | Anti-anxiety drugs | 15,463 | 445 (2.9) | 869 (5.6) |
|  | Anticonvulsants | 2,468 | 63 (2.6) | 133 (5.4) |
|  | SSRI | 574 | 15 (2.6) | 37 (6.4) |
|  | Methotrexate | 976 | 24 (2.5) | 38 (3.9) |
|  | Calcimimetics | 13 | 0 (0.0) | 0 (0.0) |
|  | Phosphate binder | 298 | 7 (2.3) | 12 (4.0) |

Data are *n* (%).

*CKD* chronic kidney disease, *COPD* chronic obstructive pulmonary disease, *SSRI* selective serotonin reuptake inhibitor
