# Supplementary figures and images for: Real-world fracture risk, osteoporosis treatment status, and mortality of Japanese non-dialysis patients with chronic kidney disease stages G3–5
Source: Clin Exp Nephrol. 2024 Oct 14;29(2):236–47. doi: 10.1007/s10157-024-02562-y (PMC11828842; doi:10.1007/s10157-024-02562-y)

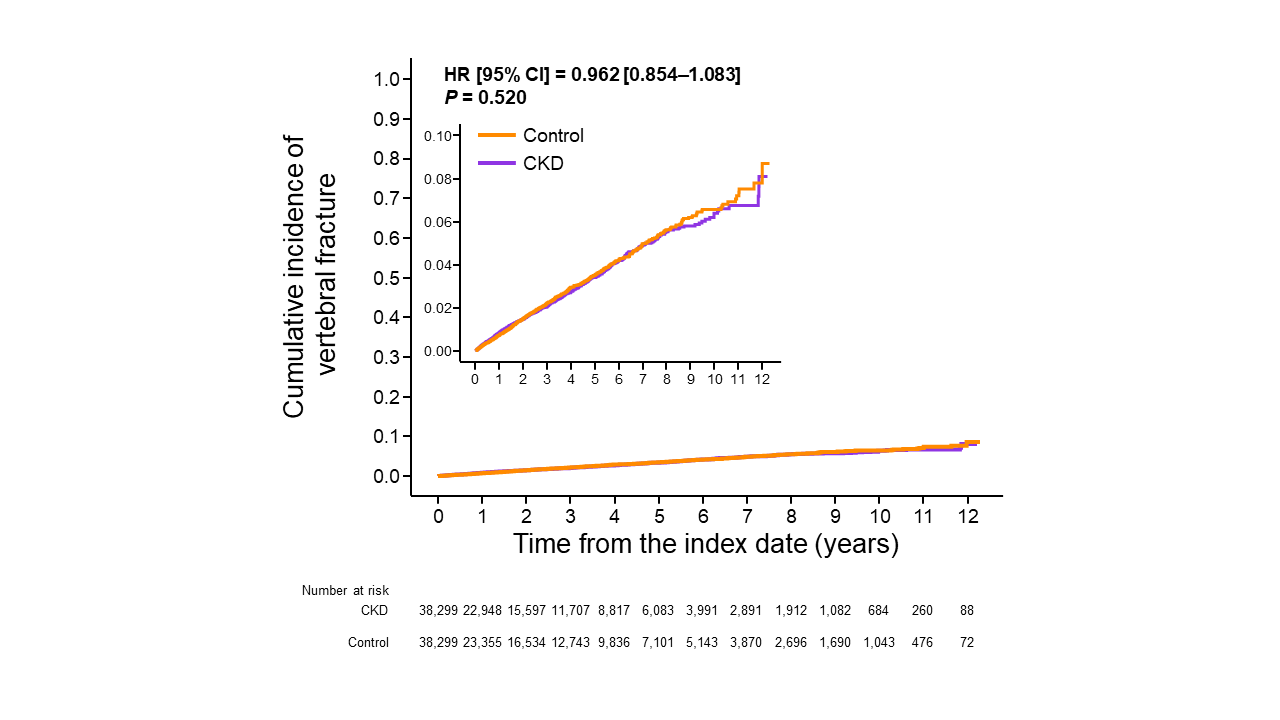

Supplement: Supplementary file 2 — Supplementary file2 (TIF 57 KB) [file 10157_2024_2562_MOESM2_ESM.tif]

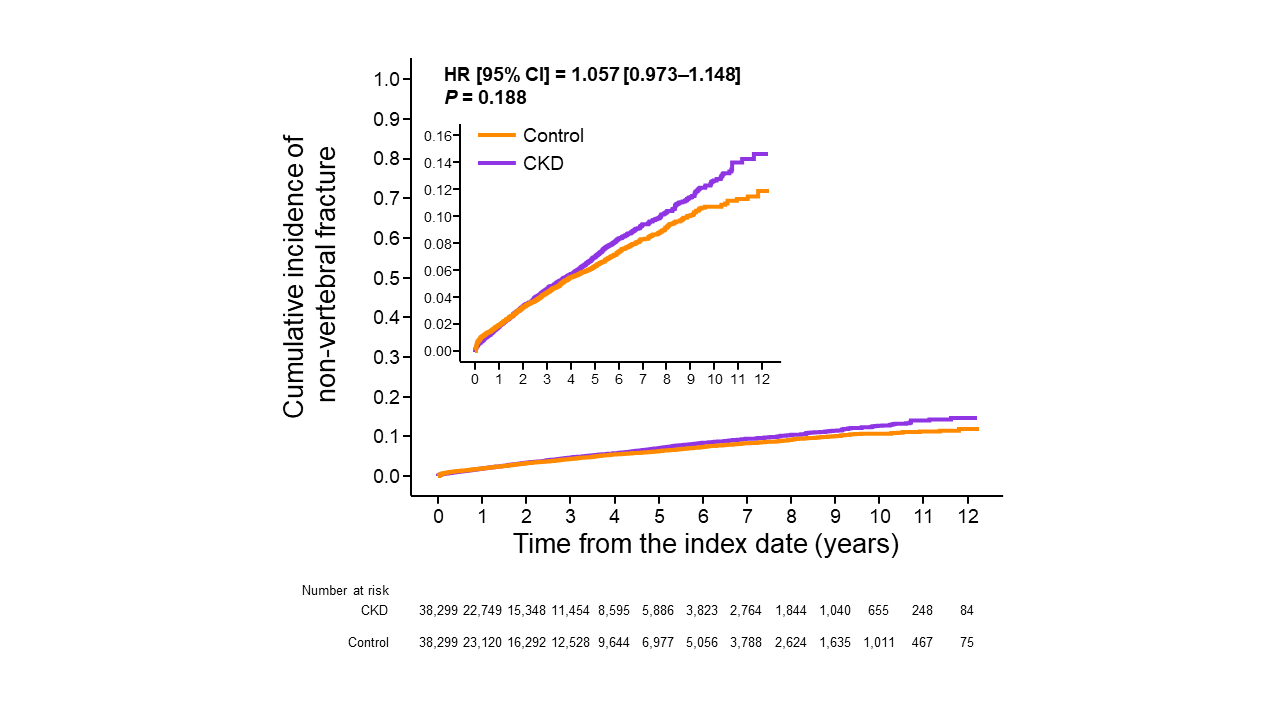

Supplement: Supplementary file 3 — Supplementary file3 (TIF 61 KB) [file 10157_2024_2562_MOESM3_ESM.tif]

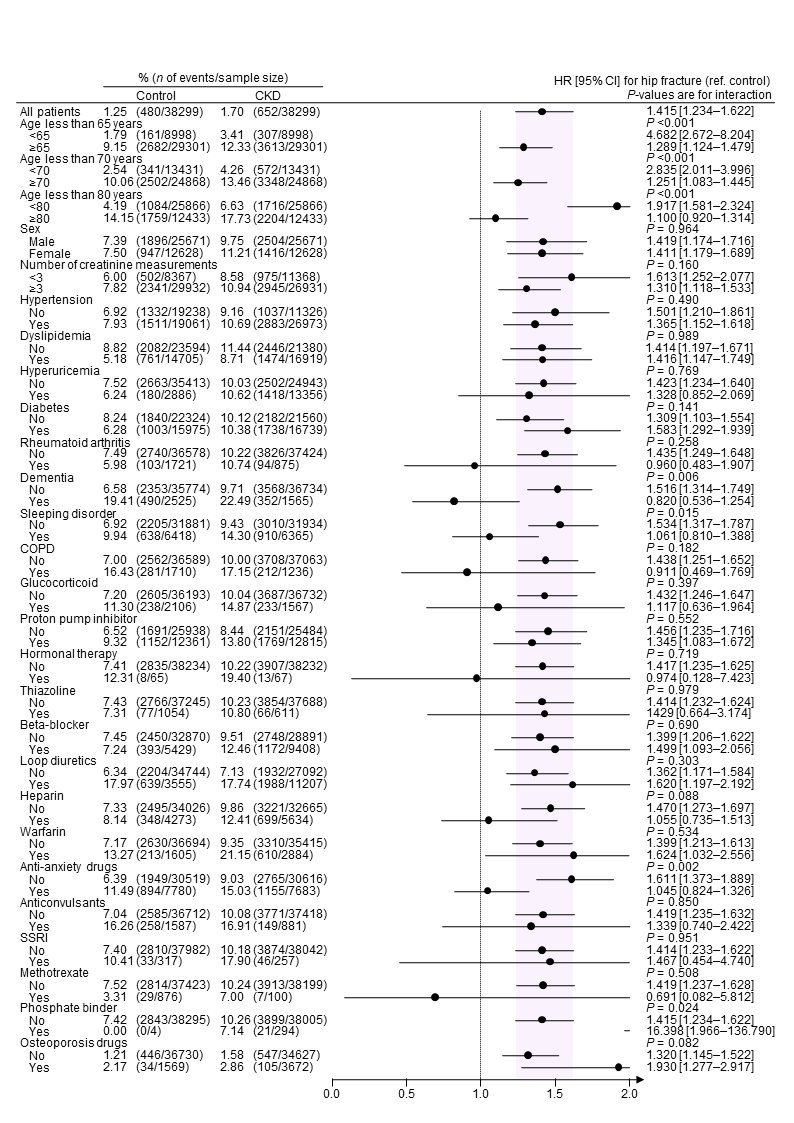

Supplement: Supplementary file 4 — Supplementary file4 (TIF 182 KB) [file 10157_2024_2562_MOESM4_ESM.tif]

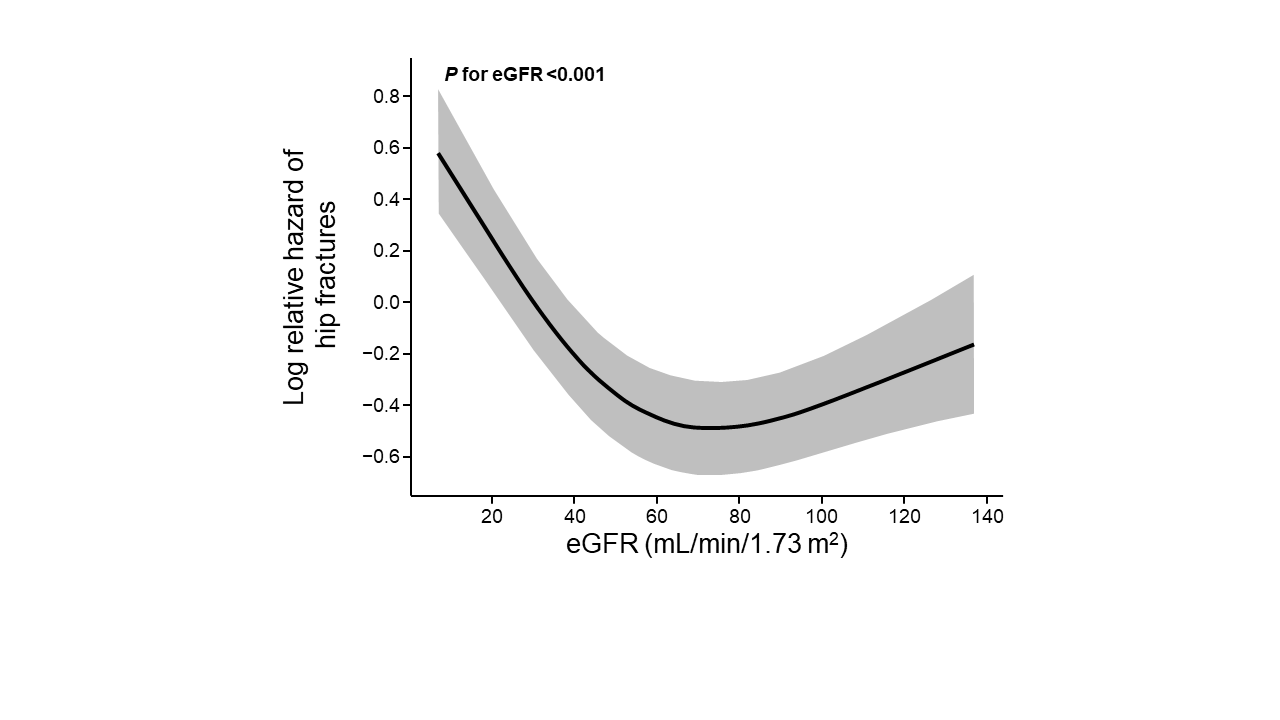

Supplement: Supplementary file 5 — Supplementary file5 (TIF 42 KB) [file 10157_2024_2562_MOESM5_ESM.tif]
